# Comparison of the Polyphenolic Profile of *Medicago sativa L*. and *Trifolium pratense L*. Sprouts in Different Germination Stages Using the UHPLC-Q Exactive Hybrid Quadrupole Orbitrap High-Resolution Mass Spectrometry

**DOI:** 10.3390/molecules25102321

**Published:** 2020-05-15

**Authors:** Elena Roxana Chiriac, Carmen Lidia Chiţescu, Daniela Borda, Mariana Lupoae, Cerasela Elena Gird, Elisabeta-Irina Geană, Giorgiana-Valentina Blaga, Rica Boscencu

**Affiliations:** 1 Faculty of Pharmacy, “Carol Davila” University of Medicine and Pharmacy of Bucharest, 37 Dionisie Lupu Street, Sector 2, 020021 Bucharest, Romania; roxana.chiriac10@yahoo.com (E.R.C.); cerasela.gird@umfcd.ro (C.E.G.); rica.boscencu@umfcd.ro (R.B.); 2 Faculty of Medicine and Pharmacy, “Dunarea de Jos” University of Galaţi, 35 A.I. Cuza Str., 800010 Galaţi, Romania; mariana.lupoaie@gmail.com; 3 Faculty of Food Science and Engineering, “Dunarea de Jos” University of Galaţi, Str. Domnească 111, 800201 Galaţi, Romania; daniela.borda@ugal.ro (D.B.); giorgianablaga89@ymail.com (G.-V.B.); 4 National Research &Development Institute for Cryogenics and Isotopic Technologies (ICSI Rm. Valcea), 4th Uzinei Street, 240050 Râmnicu Vâlcea, Romania; irina.geana@icsi.ro

**Keywords:** germination, bioactive compounds, UPLC-Orbitrap-MS/MS, MS fragmentation pattern, identification

## Abstract

Identification and quantification of polyphenols in plant material are of great interest since they make a significant contribution to its total bioactivity. In the present study, an UPLC-Orbitrap-MS/MS approach using the variable data acquisition mode (*v*DIA) was developed and applied for rapid separation, identification, and quantification of the main polyphenolic compounds in *Medicago sativa L*. and *Trifolium pratense L*. sprouts in different germination stages. Based on accurate MS data and fragment ions identification strategy, a total of 29 compounds were identified by comparing their accurate masses, fragment ions, retention times, and literatures. Additionally, a number of 30 compounds were quantified by comparing to the reference standards. Data were statistically analysed. For both plant species, the sprouts of the third germination day are valuable sources of bioactive compounds and could be used in phytotherapy and nutrition. Although *Trifolium pratense L.* (Red Clover) is considered to be a reference for natural remedies in relieving menopause disorders, alfalfa also showed a high level of biological active compounds with estrogenic activity.

## 1. Introduction

The consumption of sprouts, common in Asia, has been growing in western countries in the last decade once they were found to possess a broad spectrum of biologically active properties such as antioxidant, anti-inflammatory, allelopathic, and viewed as a valuable dietary supplement [1,2,3,4]. *Medicago sativa L* and *Trifolium pratense* sprouts are commonly consumed worldwide. They belong to the *Fabaceae* family, generally known by their commonly edible seeds [4,5,6].

Due to the increase in the use of sprouts in the human diet, there has been an expansion in the scientific literature regarding their phytochemical contents and biological proprieties. Nutritional properties of *Fabaceae* have been investigated in numerous studies [1,7].

The most interesting secondary metabolite classes found in *Medicago species* are the triterpene saponins and the polyphenolic compounds. The extraction, profiling, and identification of *M. sativa* saponins were extensively studied [8,9,10,11]. In contrast to the saponins, *Medicago* polyphenols are less genus-specific and generally encountered in many legumes. Nonetheless, alfalfa contains specific potentially valuable flavonoids with phyto-estrogenic abilities, which makes it particularly interesting for chemical characterization and pharmacological studies [12].

Red clover (*Trifolium pratense* L) sprouts are recognised as a source of phytoestrogens with high biological activity and as a dietary supplement reducing menopausal symptoms [13,14]. However, most studies focused only on certain classes of phenolic compounds such as isoflavones compounds with phytoestrogenic activity and their glycosides [13,15,16]. Among them, other polyphenolic compounds were identified by high-performance liquid chromatography HPLC in *Trifolium pratense L*.: glycitein, pratensein, pseudobaptigenin, and prunetin [16]. The comprehensive profile of phenolic compounds in aerial parts of *Trifolium pratense L*. extracts was obtained by the HPLC-tandem mass spectrometry HPLC-MS/MS technique [17,18].

Fewer reports have developed methods for qualitative and/or quantitative analysis of polyphenols in alfalfa. Flavonoids content in alfalfa was analysed in aerial parts [19,20] or in commercial sprouts by HPLC [5,6]. Ferulic acid, luteolin, myricetin, and *p*-cumaric acid were quantified in alfalfa sprouts by Oh and Rajashekar (2009) [1] using an HPLC system. However, due to the limitation of applied instrument methods, only high-level components were studied in previous studies. Moreover, a comprehensive overview of the polyphenols content variation in red clover and alfalfa during germination has not yet been reported.

High resolution mass spectrometry (HRMS), which is able to provide the accurate mass of unknown compounds, has become an important tool for characterizing chemical components in a natural product [21,22]. In the present work, we describe a comparative study conducted on alfalfa and red clover seeds and sprouts during different germination stages. Extensive characterisation of polyphenolic compounds was done by quantitative and qualitative analysis performed using ultra high-performance liquid chromatography-Q Exactive hybrid quadrupole-orbitrap high resolution accurate mass spectrometry (UHPLC-Q-Orbitrap HRMS). Thus, 29 compounds were tentatively identified without a reference standard, based on their retention times, mass spectra in a full scan mode (MS), and fragmentation patterns observed in MS-MS mass spectra. A number of 30 major compounds were unambiguously identified and quantified by comparing with reference standards. 

A fully non-targeted approach of data acquisition with and without fragmentation in one single run was developed. A full scan acquisition event without fragmentation at 70,000 full width at half-maximum FWHM of resolving power was followed by five consecutive fragmentation events at a resolving power of 35,000 FWHM (variable data independent acquisition, *v*DIA) where all ions from the full scan range are fragmented. Data Independent Acquisition is an advanced option to perform untargeted fragmentation, where the entire full scan mass range is segmented in a number of subsequent fixed m/z precursor ion ranges, which are fragmented subsequently. Thus, the fragment can be restricted to the masses in a certain fragmentation event. Zomer and Mol (2015) [23] and Elmiger (2018) [24] used this approach in the analyses of small molecules as pesticides and drugs. Compared to all-ion fragmentation, vDIA can improve selectivity because product ions result from a smaller range of precursor ions [23,24].

The data obtained were subjected to statistical processing using multivariate analysis (PCA) and hierarchical clustering analysis (HCA). The present study results could represent a novel opportunity for food science and health promotion so that only certain classes of phenolic compounds in alfalfa and red clover plants/sprouts were described in previous works [13,15,18,19]. Based on our knowledge, this is the first comparison study on the chemical profile of polyphenolic compounds in sprouts of red clover and alfalfa in different germination stages.

## 2. Results and Discussions

Both the extraction and the instrumental method used were optimized within the present study. Three different extraction procedures are tested and compared: tinctures, microwave-assisted (MAE), and ultrasound assisted extraction (UAE). The details regarding the extraction optimisation are presented in the Supplementary materials along with details on the optimisation of the electrospray ionization parameters and mobile phase selection. According to the experimental results (Supplementary Figure S1), the UAE method was subjected for further method validation.

### 2.1. Identification of Phenolic Compounds in Alfalfa and Red Clover Sprouts

Identification and quantification of polyphenols in plant material are of great interest as they make a significant contribution to its total bioactivity. A specific non-target UHPLC-Q-Orbitrap HRMS method for rapid identification of the samples components was developed, optimized, and validated. A total of 59 polyphenolic compounds were simultaneously identified including nine phenolic acids, 22 isoflavones and glucoside derivatives, 11 flavone, six flavanone, and nine flavonols. Among them, 30 major compounds were unambiguously quantified by comparing with reference standards. The retention time, compound name, formula, *m/z* values of adduct ions, and MS/MS fragment ions in negative ESI mode, mass error, and accurate molecular mass are shown in Table 1.

For the compounds without available references, the structures were presumed based on high-accuracy analysis of deprotonated precursors and fragment ions of specific components. The chemical elemental composition for each peak was assigned within a mass error of 2 ppm. Based on literature [4,12,13,17,18,20,25], a self-built chemical database of known polyphenolic compounds in seeds and sprouts was achieved. A total of 29 compounds were identified in the analysed extracts (Table 2, Supplementary Figure S2).

The fragmentation pattern of polyphenols in negative electrospray ionization has been extensively studied [26,27,28,29,30,31]. The retro-Diels–Alder (rDA) reaction, loss of a methyl radical, and the mechanism of eliminating CO, CH_2_CO, and CO_2_ at ring C are followed by successive specific fragmentations that were previously described. MS ^2^ data obtained in the present study was consistent of literature sources [31,32,33].

A number of 13 isoflavones and two isoflavones glycosides were identified and listed in Table 2. In the MS-MS spectra of the methoxylated isoflavones, the loss of the [M-H-CH_3_]^-^ radical anion, loss of a hydrogen atom in these radical anions, and the neutral losses of CO and CO_2_ were commonly observed [31].

**Figure 1:**
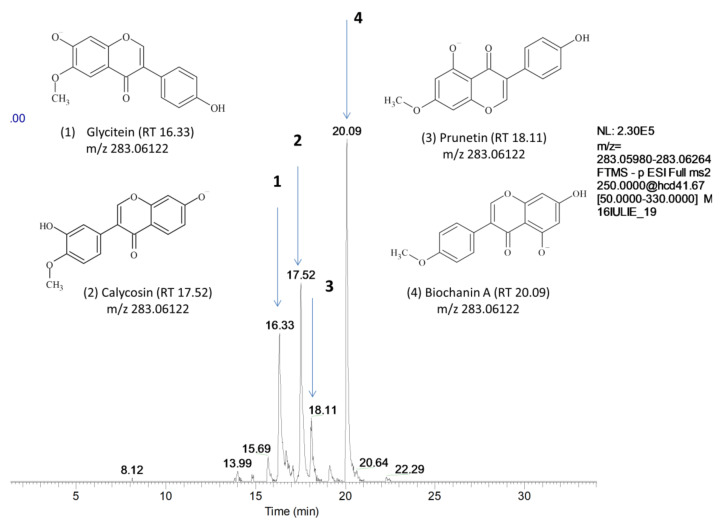
Extracted chromatograms for [M − H]^−^ 286.06, full MS ^2^ (50–330 *m*/*z*). The isomeric peaks were assigned to (**1**) glycitein, (**2**) calycosin, (**3**) prunetin, and (**4**) biochanin A.

Four isomeric peaks displayed the same [M – H]^−^ at *m*/*z* 283.06 (C_16_H_12_O_5_) in the extracted ion chromatogram of extracts of alfalfa and red clover spouts (Figure 1) and were assigned as biochanin A, prunetin, calycosin, and glycitein after the comparison with the fragmentation pattern in the mentioned databases based on the presence of some key fragments (Table 3, Supplementary Figure S3). In both alfalfa and red clover sprouts corresponding to the germination days 3 to 5, a fifth peak at *m*/*z* 283.06 was displayed at 16.58 min. (mass error 1.3) and it was assigned to 5,7-dihydroxy-2′-methoxyflavone based on the pattern fragmentation.

Glycitein, which is available as a reference standard, was identified by comparing with the retention time and fragmentation pattern of the reference solution. The fragment ion *m*/*z* 147.00 corresponding to [M-H-CH_3_-CO-B-ring]^−^ is a characteristic fragment ion of glycitein, which can be used to differentiate glycitein from its isomers and was identified only in glycitein spectra. The rest of the compounds were deduced based on the presence of diagnostic fragment ions. The loss of CH_3_· (*m/z* 268) followed by loss of a hydrogen atom (*m/z* 267) was characteristic of all four compounds. The loss of CO· moiety from demethoxylated precursors generated the ion *m/z* 240 and the loss of CO_2_ generated fragment *m/z* 224. Loss of a hydrogen atom was observed in the fragments *m*/*z* 240 and *m*/*z* 224, which both gave peaks at *m*/*z* 239 and *m*/*z* 223. Both *m/z* 240 and *m/z* 239 ions further lose CO·and CO_2_ to produce *m*/*z* 212 and *m*/*z* 211 ions, respectively, in biochanin and glycitein. In an attempt to differentiate biochanin, prunetin and isoprunetin Frański (2018) [31] showed that biochanin and prunetin cannot be differentiated by MS/MS experiments in which fragmentation occurs in a collision chamber. In the previously mentioned study, by recording full scan mass spectra at high cone voltages, differences in ions’ abundance were obtained. We tried to overlap that information on our data but the differences were not consistent and were not repeatable in real vegetal samples.

Among possible differences between those four compounds, the mass spectral decompositions in the MS2 mode concerning the retrocyclization cleavages are of special interest. The ions of type [M-H-CH_3_-CO-CO_2_-CO]^−^(*m*/*z* 168) characteristic for glycitein, [M-H-CO-B-ring]^−^(*m*/*z* 167.03) characteristic for prunetin and glycitein and [M-H-CH_3_-CO-B-ring]^−^(*m*/*z* 147) were used in tentative compound identification (Table 3). Ions [A-ring fragment]^−^(*m*/*z* 135.00) and [B-ring fragment]^−^(*m*/*z* 132.02) were characteristic for biochanin and were not detected in the rest of the isomers.

The peak at the retention time of 18.22 displayed at *m/z* 267.03 (C_15_H_8_O_5_) was deduced as coumestrol based on the presence of diagnostic fragment ions *m/z* 266.03 produced by loss of H and *m/z* 239.03, 223.04 and 211.01 (mzCloude^TM^). Biachi (2016) suggested the fragmentation pattern for coumestrol [34]. The loss of CO· moiety from the precursor ion generated the ion *m*/*z* 239.03 and the loss of CO2, the ion *m*/*z* 223. The *m/z* 239.03 ion further lose CO·and CO_2_ to produce *m*/*z* 211.04 and *m*/*z* 167.10 ions, respectively. All fragments were identified in the alfalfa extracts samples.

**Figure 2:**
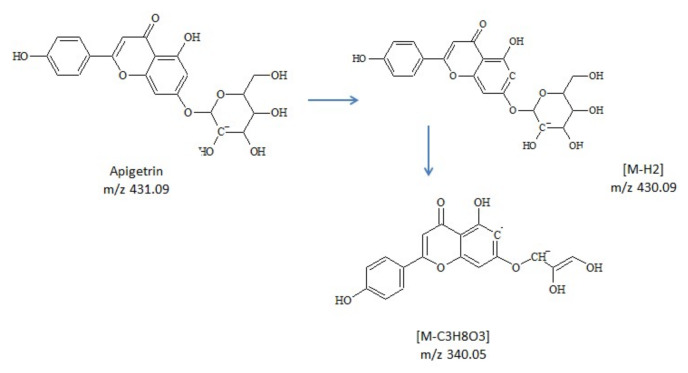
Proposed pathway for fragment *m*/*z* 340.05, specific for apigetrin.

At 12.46-min and 22.02-min hexose glycoside of coumestrol and, respectively, biochanin A were identified. The loss of sugar moieties was observed, producing a fragment ion [M-H-Glc]^−^ corresponding to the aglycone form. For apigenin glucoside, two isomers were observed and assigned as vitexin (apigenin8-*C*-glucoside) and apigetrin (apigenin-7-glucoside). The fragmentation of each isomer was predicted using MS Fragmenter software. Due to their structural differences, fragment *m*/*z* 340.05 [M-C_3_H_8_O_3_]^−^ was specific only for apigetrin (Figure 2).

Peaks observed at the retention time 15.30 and 18.1921 minutes displayed the same exact mass *m*/*z* 256.0735 and was assigned as liquiritigenin and isoliquiritigenin (C_15_H_12_O_4_) in reference to the mass spectral database and the previously reported study [33,34]. The diagnostic ions were *m*/*z* 135.00, 119.04, which were consistent with typical [^1,3^A − H]^−^ and [^1,3^B − H]^−^ fragments [33].

Three isomeric peaks with the same [M − H]^−^ ion at *m*/*z* 299.06 (C_16_H_12_O_6_) displayed at 15.24, 18.20, and 19.70 min in the extracted ion chromatogram of alfalfa samples and were assigned to chryosoeriol, tectorigenin, and pratensein based on literature [12,15] and fragmentation pattern (Supplementary Figure S4). The fragments at m/z 284.03 formed by demethoxylation and m/z 256.06 generated by the future loss of CO were characteristic for all three compounds (Table 4). Fragment ions at *m*/*z* 267.03 [M-H-HO-CH_3_]^−^ and 255.06 [M-H-CO_2_]^−^ are also detected in the MS2 spectrum of tectorigenin. The minor fragment ion at *m*/*z* 135 [M-H-Aring]^−^ correspond to the cleavage of the B ring pathway. The results were consistent with previous studies [35,36]. The formation of an ion at *m*/*z* 151.04 comprising the ring A of the flavone skeleton was also c haracteristic for the mass spectral decomposition in tectorigenin.

The extracted chromatogram for *m*/*z* 285.04 [M − H]^−^ in the red clover spout extracts revealed two peaks at 11.74 min 17.06 min. The last one was identified as kaempferol according to the retention time and fragmentation pattern of the reference standard. According to the fragment interpretation using MS Fragmenter software and the published data, the first peak was identified as baptigenin. In the MS/MS, spectrum were detected signals at *m*/*z* 269.04 corresponding to the [M -OH]^−^ fragment, *m*/*z* 240.04 as [M-H-CO_2_-H]^−^, *m*/*z* 136.01 as [M-B-ring-C_2_H]^−^, and 109.02 corresponding to the [B ring]^−^.

Peck at [M − H]^−^ *m*/*z* 297.04 (RT 17.21) was identified in the red clover sprouts samples as irilone, which is in agreement with other studies [25,37]. The diagnostic ions were: *m*/*z* 269.04 corresponding to [M-H-CO]^−^, *m*/*z* 252.04 [M-H-CO_2_-H]^−^, *m*/*z* 178.99 [M-H-B ring-CO]^−^, *m*/*z* 133.02 [M-C_8_H_5_O_4_]^−^. Isoflavone pseudobaptigenin [M − H]^−^ *m*/*z* 281.04 (RT 21.29) was identified in both alfalfa and red clover sprout samples based on the diagnostic ions *m*/*z* 285.05 [M-H-CO]^−^, *m*/*z* 251.03 [M-H-CO-H_2_]^−^, and *m*/*z* 135.00 [M-C_9_H_7_O_2_]^−^. Azelaic acid, [M − H]^−^ at *m*/*z* 187.09 (RT 15.11), which is naturally occurring in wheat, rye, barley, oat seeds, and sorghum, was identified in all analysed samples based on the diagnostic ions *m*/*z* 171.10 [M-OH]^−^, *m*/*z* 125.09 [M-CH_3_O_4_]^−^, and *m*/*z* 123.08 [M-CH_4_O_3_]^−^, according to the MS Fragmenter Software.

The other compounds listed in Table 2 were identified according to their molecular mass, formula, MS/MS fragments, and related literature by using the same approach. Regarding those compounds, distribution in the analysed samples and the variation during germination is shown in Supplementary Figure S5.

The isoflavone aglycones biochanin A, pseudobaptigenin, calycosin, prunetin, and pratensein previously reported in the red clover aerial part [16,18,19] were detected in both red clover and alfalfa seeds and sprouts. Comestrol, tricin, vitexin (apigenin 8-C-glucoside), and tectorigenin were found only in alfalfa sprout samples, while afrormosin, baptigenin, and irilone were detected only in red clover samples. Afrormosin and irilone appear due to various modifications of the isoflavones A-ring and have been reported in aerial parts of plant form *Fabaceae* family [25].

Although biochanin A, formononetin, genistein, daidzein, and their glycosides are commonly determined in red clover as phytoestrogens of interest [18,38,39], other compounds identified in the present study that may contribute to the significant estrogenic activity of red clover include medicarpin, liquiritigenin, and isoliquiritigenin [19]. All of these compounds were found both in alfalfa and red clover sprouts.

Chrysoeriol and its glycoside, previously reported in the aerial part of alfalfa [20,38], were identified in both species of spouts. Medicarpin and chrysoeriol show significant antiangiogenic and cytotoxic activity [40] suggesting an interesting potential in cancer therapy and justify further studies.

Coumestrol was identified only in alfalfa sprouts. However, alfalfa sprout was recognised as a major source of coumestrol [19]. Tricin was detected in all samples of alfalfa seeds and sprouts, which is consistent with other studies reporting tricin identification in the alfalfa aerial part [12]. Luteolin-7-*O*-glucoside, kaempferol-3-*O*-glucoside, kaempferol-3-*O*-rutinoside previously reported in red clover, alfalfa, and mung bean sprouts [6] were identified in both species in different germination stages.

The compounds identified in sprouts’ samples exhibit a wide range of biological effects, including antioxidant, antimicrobial, phytoestrogenic effects, and anticarcinogenic activity [13,15,19,40]. Our study findings confirm that both alfalfa and red clover sprouts are important sources of isoflavones besides soy and soy-derived products. Furthermore, according to the literature, while widely used soy is a source of poorly absorbed isoflavones glycosides, red clover and alfalfa contains easily-absorbed free aglycones forms [5].

### 2.2. Quantification Result

The developed UPLC-HRMS/MS method was applied for the routine determination of 30 compounds in the extracts of seeds and sprouts of alfalfa and red clover identified in Table 1a. The method was validated according to the section “Quantitative Method validation.” Validation parameters are provided in Supplementary Table S1. The result of the quantification of the target compounds in alfalfa and red clover seeds and sprouts were shown in Table 5 and Table 6. The analysis was conducted in duplicate. The obtained data were expressed as mean values ± standard deviations.

Among the quantified compounds, ononin, naringin, and epicatechin levels were below quantification limits. The results of the quantitative analysis are consistent with other studies [6,18,39]. Germination is a process known to be accompanied by a spectrum of significant changes in metabolites. Phenolic compounds are already present in the earliest plant stages and have crucial functions in plants’ evolution and adaptation. While the total polyphenols’ content varies, there are some kinetic transformations of the individual components that are directed towards reducing or increasing the activity over time to support specific metabolic pathways [25].

The quantitative results showed a high content in catechin, hesperetin, and quercetin in red clover seeds. The content in catechin sharply decreased after the first day of germination to a value almost six-fold lower and it continued to be reduced over time, up 20-fold on the fifth day of red clover germination. Quercetin concentration was four times lower on the fifth day of germination when compared to red clover seeds. The concentration of hesperetin in the red clover seeds linearly decrease 3.47-fold from the first to the fifth day of germination. Rather, rutin concentration increased during germination, reaching the maximum concentration on the fifth day of germination.

Respecting the alfalfa seeds and sprouts, lower variation of compounds’ concentrations was registered for the sprouts in the second, third, and fourth day of germination. However, clear differences were observed between the seeds and spouts. For example, the considerably higher concentrations of kaempferol, quercetin, syringic acid, and hyperoside were measured in the seeds. Hesperitin dynamics during germination was reversed when compared to red clover, registering a marked increase in the third day of germination, which is followed by a slight decrease.

The dynamics of ferulic acid and *p*-coumaric acid concentration during germinations was similar in alfalfa to the one in red clover, with higher concentrations of *p*-coumaric acid and lower concentrations of ferulic acid in alfalfa than in red clover during germination. *p*-coumaric acid can be converted into caffeic acid via hydroxylase activity during plant germination [25]. According our results, *p*-coumaric acid showed a similar decrease during germination for both species. In alfalfa sprouts, almost double the amount of *p*-coumaric acid was measured when compared to red clover. However, caffeic acid was detected only in red clover samples, which suggests different metabolic pathways. The highest concentrations of caffeic acid were measured in red clover seeds and in sprouts on the third day of germination.

The profile of the isoflavones with estrogenic activity varied greatly with the phenological stage along the germination. In the red clover sprouts, aglycones daidzein and genistein concentrations increased from the first to the fourth day of germination, with a maximum on the third day, while, on the fifth day, the concentration was considerably lower. The concentration of formononetin in red clover increased during germination 12-fold up to day 4 when compared to the seeds. Glycitein was found in the highest concentration on the third day of germination.

Formonetin and glycitein concentrations in alfalfa sprouts on the third germination day were comparable with red clover sprouts in the same germination stage, while the concentrations in genistein, genistin, daidzein, and daidzin were considerably lower. However, other compounds that manifest an estrogenic effect include myricetin and apigenin [41]. Apigenin was found only in alfalfa sprouts with an increasing concentration up to three times higher in sprouts on the third day when compared to seeds. Apigenin was previously reported in alfalfa aerial parts [20,42]. Myricetin concentration in the alfalfa sprouts on the third germination day was about 10-fold higher than in red clover sprouts.

In contrast to the red clover sprouts, which contain genistein as the major isoflavons, in alfalfa sprouts, the most abundant isoflavone was formononetin, which is consistent with other studies [37]. Genistein content in alfalfa and red clover spouts, reaching the maximum level on the third day of germination (607.2 µg/g for red clover and 105.8 µg/g for alfalfa), might be considered high, even compared to soybean in which genistein was reported as the major isoflavone, ranging from 84 µg/g to 583 µg/g DW along the reproductive stages [42]. According to the quantitative analysis, the sum of the isoflavones with estrogenic activity (daidzein, genistein, glycosides, apigenin, formononetin, myriceitin, and glycitein) was 906.1 µg/g DW for red clover and 587.5 µg/g DW for alfalfa sprouts on the third day of germination. Due to the different compounds’ bioavailability [43], consideration of their biological activity could be speculative.

While rutin concentrations in all red clover samples were significantly higher than in alfala, comparable concentration of naringenin, syringic acid, ellagic acid, isorhamnetin, and pinostrobin were measured in both species during germination. For isorhamentin, the same nonlinear variation was observed for both species with the maximum concentration reached on the fourth day of germination. Isorhamnetin 3-*O*-glucoside was also identified in both species. Isorhamnetin has been previously detected, but not quantified in alfalfa aerial parts [38].

Abscisic acid, which is a plant hormone that has important roles in seed development and maturation [44], was present in relatively small amounts in both red clover and alfalfa in seeds and during germination.

In conclusion, consider the quantitative results for both plants species. Sprouts on the third day of germination could be considered as valuable sources of bioactive polyphenols with a potential health impact. The important health promoting potential of polyphenols resides in various biological activities, including antioxidant, estrogenic, anti-carcinogenic, and vasodilatory [16,25,37]. Different studies described the interactions of phenolic derivatives with intracellular receptors and signaling pathways to induce adaptive responses and regulation of apoptotic genes and mitochondrial function [3,15]. They suggest that *Fabaceae* sprout consumptions may also reduce the risk of osteoporosis, help alleviate menopausal symptoms, and prevent cardiovascular disease, hypertension, and hormone-dependent tumours [5,41].

### 2.3. Multivariate Data Analysis

**Figure 3:**
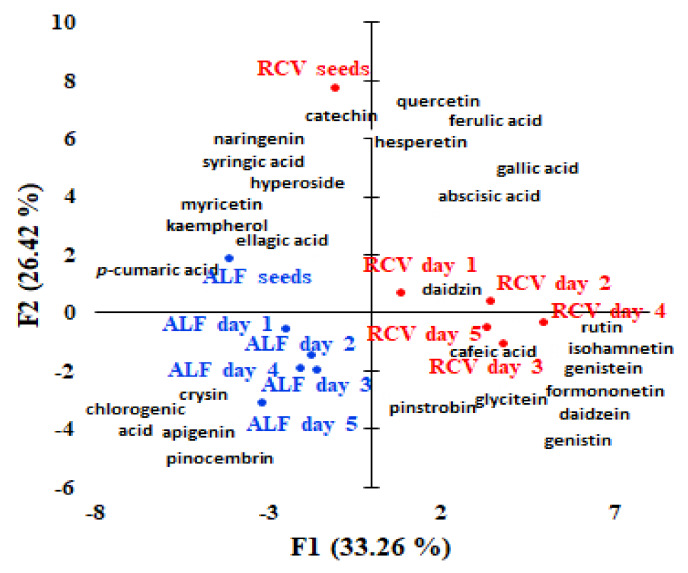
Bi-plot of the principal components PC1 and PC2 resulted from the PCA analysis with normalized Quatrimax rotation data of the polyphenols’ concentrations quantified and the samples (alfalfa and red clover sprouts samples coded as: RCV — red clover, ALF — alfalfa, s — seeds, day 1-first day of germination, day 2-second day of germination, day 3-third day of germination, day 4-fourth day of germination, and day 5-fifth day of germination).

Unsupervised classification by PCA and HCA were used in order to show the grouping of the investigated samples. PCA explained 59.68% of the total variation using principal components with a higher contribution brought by PC1 (33.26%) when compared to PC2 (26.42%), (Figure 3). Along PC1, the germinated alfalfa and red clover seeds from the initial seeds can be discriminated. Along the PC2 axis, two clusters including the firs cluster including alfalfa seeds and germinated alfalfa seeds located on the left side and cluster of two grouping the germinated red clover seeds located on the right side are observed. Non-germinated red clover seeds are clearly discriminated from the other seeds. PCA analysis revealed the correlations among the polyphenols’ composition of different germinated seeds. Our results showed that ellagic and *p*-coumaric acids, kaempferol, and myricetin represent polyphenol markers of alfalfa seeds, while chlorogenic acid, chrysin, apigenin, and pinocembrin are representative for germinated alfalfa seeds. Red clover seeds were characterised by catechin, naringenin, hyperoside, and syringic acid, while gallic and ferulic acids, quercetin, hesperitin, and abscisic acid characterise the red clover seeds on the first and second day of germination and caffeic acid, pinostrobin, glycitein, rutin, isorhamnetin, genistein, formonetin, daidzein, and genistin are representative of the germinated red clover seeds on day 3, day 4, and day 5.

**Figure 4:**
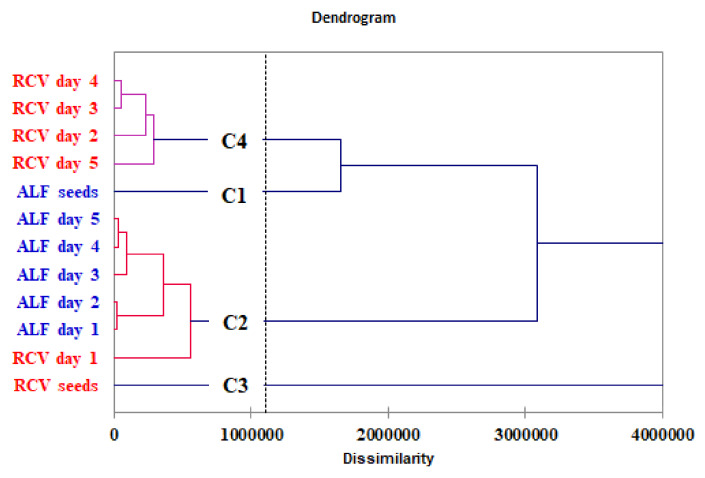
Dendrogram of the 12 objects (alfalfa and red clover sprout samples) represented by phenolic compounds’ profile obtained by Ward’s hierarchical clustering method (hierarchical cluster analysis).

The Ward’s hierarchical clustering method with Euclidean distances as measures of dissimilarity based on the phenolic compounds’ profile was applied. The dendrogram shows the clustering of the samples in four separate groups (Figure 4). At a dissimilarity level of 1200000, class 1 refers to the initial alfalfa seeds (ALF seeds) (C1), while class 2 (C2) include the germinated alfalfa seeds in the five days of germination and red clover seeds on the first day of germination. Class 3 (C3) refers to the initial red clover seeds (RCV seeds) and class 4 (C4) correspond to the germinated red clover seeds, which reveal a polyphenolic composition similar to the initial alfalfa seeds.

**Figure 5:**
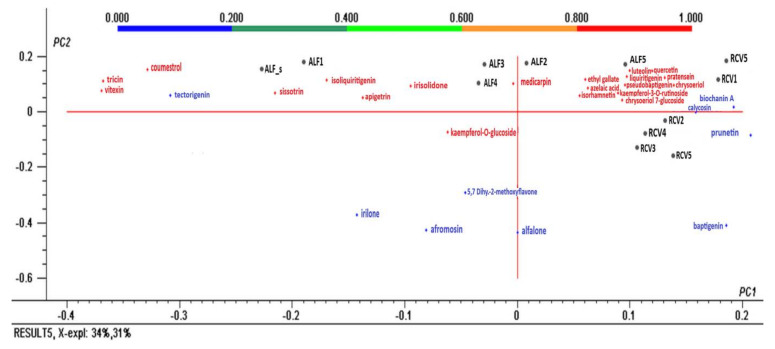
Bi-plot of the principal components PC1 and PC2 resulting from the PCA analysis with normalized Quatrimax rotation data of the tentatively identified polyphenols and the samples.

The second PCA-analysis (Figure 5) was performed for the results of the qualitative analysis: identified polyphenols obtained from the screening HRMS/MS ^2^ and indicated with a present / absent decision.

PCA analysis revealed the correlations among the polyphenols’ composition of different germinated seeds. Thereby, coumestrol, tricin, vitexin, tectorigenin, and sissotrin represent polyphenol markers of alfalfa seeds, while isoliquiritigenin, apigetrin, irisolidone kaempferol-*O*-glucoside, medicarpin, irilone, alfalone, and afrormosin are representative for germinated alfalfa seeds. Azaleic acid, isorhamnetin, luteolin-7-glucoside, liquitrigenin, pratensein, pseudobaptigenin, chrysoeriol, chrysoeriol-7-glucoside, and kaempferol-3-rutinoside were characteristic for alfalfa in the five days of germination, while biochanin A, calycosin, prunetin, and baptigenin are characteristic for red clover.

## 3. Materials and Methods

### 3.1. Reagents

The reference standards of 30 compounds (bold in Table 1) were purchased from Sigma–Aldrich (Aquator, Iasi, Romania). Organic solvents’ methanol and ethylic alcohol, HPLC grade, were purchased from Merck Romania. Formic acid (98%) was ultrapure water (LC-MS grade) and was purchased from Merck (Merck Romania, Bucharest, Romania). For the calibration of the mass spectrometer, the Pierce^TM^ LTQ Velos electrospray ionization (ESI) positive and negative ion calibration solutions (Thermo Fisher Scientific) were used.

### 3.2. Stock Solutions

The stock standard solutions of the reference standards were dissolved in methanol with a concentration of 1.0 mg/mL for each compound, respectively. A series of working standard solutions (concentrations ranged from 0.05 to 1.0 µg/mL) were prepared by the successive dilution of the mixture of standard solutions with 20% methanol. All the solutions were stored at 4 °C before use.

### 3.3. Plant Samples – Germination

The seeds of *Trifolium pratense* and *Medicago sativa* were purchased from Agrosem, Targu Mures, Romania. Approximately 50 g of seed of each plant species were washed in a plastic container with 20 °C sterile distilled water for 30 min and then transferred to a growth chamber (automat sprout germinator, Biovita model GE-1, Cluj-Napoca, Romania) controlled at 25 °C and 80% humidity in the dark condition. Seed hydration was automatically controlled as follows. The seeds were soaked for 7 h. Then they were irrigated with water every 5 min for 7 h, and then they were irrigated for 1 min every 4 h. For both plant species, we obtained sprouts at 24 h, 48 h, 72 h, 96 h, and 120 h. The entire sprouts were dried for 4.5 h at 40 ° C in a fruit dryer (Zilan model- ZLN-9645l). Average humidity loss was 90% for alfalfa and 89% for red clover. After drying, the twins were kept in closed containers, away from light. Before analysis, aliquots of 5 g of each sample were ground.

### 3.4. Extraction

Three extraction procedures were compared in the present work, maceration, ultrasound assisted extraction (UAE), and microwave assisted extraction (MAE). The extracts were prepared in the same condition for both plant species.

A tincture was prepared according to EU Pharmacopeia using 70% ethanol (1:10 g DW/ g). The solvent was initially heated at 60 °C. The maceration continued for 10 days in dark conditions. 

An ultrasonic assisted extraction (UAE) method optimised by response surface methodology (RSM) [45] was adapted for the present study. The extraction conditions were a ratio of liquid to solid of 1:10 g DW/g 70% ethanol, 60 °C, 60 min, at 60 kHz.

A microwave assisted extraction (MAE) procedure was adapted after Zhang, 2008 [26]. An amount of 0.5 g of each sprout sample were extracted with 15 mL and 50% ethanol (1:25 g DW/g) at 50 °C (10 min. gradient with 5 min. maintaining), and microwave power at 300 W.

For all procedures, the extracts were filtered through Whatman No. 2 filter paper and a 0.20 nm Millipore MF syringe filter. Dilution 1:3 with water:methanol (80:20) before instrumental analysis was completed.

### 3.5. Instrumentation

#### 3.5.1. LC Parameters

A Thermo Scientific Dionex Ultimate 3000 Series RS pump coupled with a Thermo Scientific Dionex Ultimate 3000 Series TCC-3000RS column compartments and a Thermo Fisher Scientific Ultimate 3000 Series WPS-3000RS autosampler controlled by Chromeleon 7.2 Software (Thermo Fisher Scientific, Waltham, MA and Dionex Softron GMbH Part of Thermo Fisher Scientific, Germany) were used for analysis.

The application of a 35-min gradient over an ultra-performance Accucore U-HPLC Column C18 (150 × 2.1 mm, 2.6 µm), (Thermo Scientific) was applied. The column temperature was set at 40 °C. The mobile phase consisted of: eluent A, ultrapure water containing 500 µL/L formic acid (pH 2.5), and eluent B, methanol. The step gradient was as follows: 0–1 min 100% A, 1–10 min linear increase to 30% B, 10–26 linear increased to 100% B and held for 4.0 min, 30–32.5 decreasing to 0% B. The initial conditions were obtained again at the 35th min with an equilibration time of 2.5 min. The run was performed at 0.4 mL/min for a total of 35 min.

#### 3.5.2. MS Parameters and Data Processing

A HESI (Heated Electrospray) ion source was used for the ionization. The HESI parameters were optimized as follows. Nitrogen as sheath and auxiliary gas flow rate was set at 8 and respectively 6 units. The source heater temperature was set at 300 ℃. The capillary temperature was set at 300 °C. The aux gas heater temperature was set at 300 °C. The electrospray voltage was 2800 V. The S lens RF level was 50.

Detection of the compounds was performed using an Q-Exactive mass spectrometer. Full scan data in negative mode was acquired at a resolving power of 70,000 FWHM at *m*/*z* 200. For the compounds of interest, a scan range of *m*/*z* 100–1000 Da was chosen. The automatic gain control (AGC) was set at 3e6 and the injection time was set to 200 ms. The scan rate was set at 2 scan/sec. External calibration was performed by calibration solution in a positive and a negative mode.

A total of six scan events were combined including one full scan event with mentioned parameters and five MS-MS events. In the MS^2^ scan events, the precursor ion ranges were *m*/*z* 95–205, 195–305, 295–405, 395–505, and 500–10005, which were consecutively selected, fragmented in an higher-energy collisional dissociation cell HCD, and measured in five separate Orbitrap scans at a resolving power of 35,000 FWHM. The fragmentation events were performed at 30, 60, and 80 NCE (normalised collision energy). The C-trap parameters for all scan events were the following: Automatic Gain Control (AGC) target 1e6 and the injection time of 100 ms.

Data were evaluated by the Quan/Qual Browser Xcalibur 2.3 (Thermo Fisher). The mass tolerance window was set to 5 ppm for the two analysis modes. For the MS/MS analysis, detection of at least two fragment ions with the appropriate ion-ratio was performed by comparing the reference standards. 

For those compounds without available references, the most reasonable molecular formula with a lower mass error was sought in the chemical Chemspider database (www.chemspider.com). Considering that the flavones, isoflavones, and phenolic acids had the same skeleton, the fragment ions from MS-MS analysis were used to further confirm the chemical structure with the aid of NORMAN MassBank (https://massbank.eu/MassBank/), mzCloude^TM^ Advanced Mass Spectral Database (https://www.mzcloud.org/), and PubChem (https://pubchem.ncbi.nlm.nih.gov/). ACDLabs MS Fragmenter 2019.2.1 software was used to generate a fragmentation pattern of the identified compounds for a comparison analysis.

#### 3.5.3. Quantification Method Validation

In the full scan mode, the accurate mass of the precursor ion was used for quantification. Five-point calibration curves were obtained with a mixed standard solution in the concentration range of 25 to 2500 ng/mL. Linearity was evaluated using the coefficients of correlation (R^2^). The LOD (limit of detection) and LOQ (limit of quantification) were determined as 3.3 times and 10 times, respectively. The standard deviation of the *y*-intercept divided by the slope of the calibration curve (ICH Validation of analytical procedures: text and methodology Q2(R1), International Conference on Harmonization, 2005) [46]. The UHPLC-Q-Orbitrap mass spectrometry was validated with respect to specificity, linearity, sensitivity, and reproducibility (ICH guidelines [46]).

The precision of the established method was evaluated by intra-day and inter-day variability, and the relative standard deviations (RSD) were taken as a measure. The RSDs were used as a measure and the acceptance criterion should be within 5.0%. The intra-day and inter-day variability were measured to assess the precision of the developed method using samples of alfalfa and red clover sprouts extract in the second day of germination. The intra-day precision was evaluated by analyzing six replicates prepared from the mentioned samples, and the inter-day precision was examined over three consecutive days with six samples per day. The repeatability was determined by injection of six samples prepared by following the same procedure.

#### 3.5.4. Multivariate Data Analysis

Principal component analysis (PCA) is a multidimensional scale analysis that enables transformation of the variables into new ones, called principal components. The role of principal components is to explain the maximum amount of variance with the fewest number of components. PCA was performed for the quantitative and qualitative matrix containing 12 samples and 25 phenolic compounds. The PCA was applied for the quantitative correlation matrix using unit vector normalization and to the binary matrix for the qualitative analysis. Quatrimax rotation was performed and the bi-plots were selected for the visualization of the results.

Additionally, the Ward’s hierarchical clustering method with Euclidean distances as measures of dissimilarity were applied.

## 4. Conclusions

In this paper, qualitative and quantitative analyses were combined together for the integrated characterisation and comparative analysis of the polyphenolic profile of *Medicago sativa L*. and *Trifolium pratense L.* sprouts in different germination stages. A variable data independent acquisition (vDIA) approach was used, which improved both selectivity and sensitivity for the fragment ions. This was beneficial for screening performance and identification capabilities.

By comparing MS/MS fragmentation patterns of reference compounds and the systematic identification strategy, a total of 59 polyphenolic compounds including isoflavones, flavones, flavonones, flenolic acids, and flavonols were identified in the alfalfa and red clover sprout extracts. A quantitative determination method had been validated and applied for the quantification of 30 compounds. Three extraction methods were optimised and compared.

The 29 phenolic compounds that have been identified in sprout extracts are: isoflavones with estrogeninc action as biochanin A, coumestrol, prunetin, isoflavones as irilone, pratensein, pseudobaptigenin, flavone as tricin, chriosoeriol, and phenolic acid as ethyl gallate. Glucosides of apigenin, kaempherol, and coumestrol or isorhamnetine were also identified. For both plant species, sprouts in the third and fourth germination days were found to contain higher quantities of biologically active isoflavones as genistin, daidzein, formononetin, glycitein, apigenin, hesperetin, quercetin, ferulic acid, and *p*-coumaric acid.

The method presented in this paper has been demonstrated as an effective pathway for analysing the bioactive compounds in a complex sample from a natural resource as sprouts of alfalfa and red clover. This study also demonstrated the feasibility and advantage of the *v*DIA strategy on untargeted screening. The development of advanced methods for analysis of individual, biologically-active compounds will enable future understanding of their mechanisms of action on human organisms.

Despite the well-known medicinal properties of *M. sativa* and wide consumption of alfalfa sprouts, only a few reports on biological activity of single compounds have been published. This study provides an important scientific basis for further study on clinical application and functional food of alfalfa and red clover sprouts.

## Figures and Tables

**Table 1:** The 30 compounds identified from alfalfa and red clover sprouts by UHPLC-Q-Exactive with structures confirmed by comparison with reference standards.

Compound Name	R.T. (min)	Formula	Exact Mass	Error (ppm)	Adduct Ion (*m*/*z*)	MS ^2^ Fragments (*m*/*z*)
**Flavonoids (flavan-3-ols, flavone, flavonols, flavonone, flavone glucoside)**						
Catechin	7.8	C_15_H_14_O_6_	290.07904	1.47	289.07176	245.08192; 203.07088; 151.03908; 125.02320; 109.02821
Epicatechin	10.19	C_15_H_14_O6	290.07904	1.25	289.07176	245.08192; 203.07088; 151.03908; 109.02821
Quercitin	16.59	C_15_H_10_O_7_	302.04265	0.86	301.0354	245.04601; 178.99809; 273.04059; 121.02814
Rutin (quercetin3-rutinoside)	14.20	C_27_H_30_O_16_	610.15338	0.5	609.14613	300.02777; 271.02505; 255.02995; 243.02980; 165.01841;151.00258
Apigenin	17.54	C_15_H_10_O_5_	270.05282	1.18	269.04502	227.03389; 181.06430; 151, 00194; 149.002266; 117.03271
Kaempferol	17.06	C_15_H_10_O_6_	286.04774	0.57	285.04049	255.02977; 201.01866; 151.00262; 107.01250; 92.9266
Isorhamnetin	13.20	C_16_H_12_O_7_	316.0583	1.35	315.05105	300.0271; 227.03508; 163.00369; 151.00264; 107.01190;
Naringenin	19.69	C_15_H_12_O_5_	272.06847	0.5	271.06122	253.05055; 151.00269; 119.04903; 107.01258
Naringin	14.11	C_27_H_32_O_14_	580.1792	2.02	579.17185	356.99371; 255.02995; 119.04884
Hesperitin	16.83	C_16_H_14_O_6_	302.07904	1.34	301.07179	283.06204; 267.06650; 252.04286; 151.00266; 125.02319
Pinostrobin	17.40	C_16_H_14_O_4_	270.08921	1.89	269.08196	254.05864; 210.06839; 177.05495; 148.01559
Pinocembrin	18.24	C_15_H_12_O_4_	256.07356	1.04	255.06631	239.0713; 237.0557; 227.0713; 179.0349; 147.0451
Chrysin	17.63	C_15_H_10_O_4_	254.05791	1.65	253.05066	208.96011; 151.03899; 107.04897; 89.04897; 65.03819
Myricetin	10.42	C_15_H_10_O_8_	318.03757	1.37	317.03032	178.9986; 164.92636; 151.00368; 137.02442; 107.01258
Galangin	19.98	C_15_H_10_O_5_	270.05282	1.48	269.04557	239.03345; 227.03389; 225.05580; 211.03877; 169.06425
Hyperoside (quercetin 3-galactoside)	13.98	C_21_H_20_O_12_	464.09548	1.03	463.08768	300.02771; 355.02985; 271.02491; 243.02969; 178.99773; 151.00262
**Isoflavone**						
Genistin	14.77	C_21_H_20_O_10_	432.10565	1.45	431.09837	311.05637; 269.04590; 271.05133; 181.06580
Genistein	18.07	C_15_H_10_O_5_	270.05282	1.24	269.04502	159.04420; 133.02835; 201.05527; 181.06546; 107.01257
Daidzin	11.42	C_21_H_20_O_9_	416.11073	1.49	415.10348	252.0451; 251.0349; 224.0487; 223.0398
Daidzein	16.50	C_15_H_10_O_4_	254.05791	0.87	253.05066	226.05887; 224.04649; 209.06091; 197.06055; 135.00686; 117.03333
Ononin	26.14	C_22_H_22_O_9_	430.12638	2.06	429.11913	355.0969; 341.1109; 267.1028; 252.00778
Formononetin	18.74	C_16_H_12_O_4_	268.07356	1.19	267.06631	252.04298; 223.03986; 195.04466; 132.02049
Glycitein	16.33	C_16_H_12_O_5_	284.06847	1.18	283.06122	268.0375; 240.0483; 211.03979; 196.05252; 167.02063
**Phenolic Acid**						
Gallic Acid	1.73	C_7_H_6_O_5_	170.02152	0.16	169.01427	125.02318; 141.01823
Chlorogenic Acid	8.20	C_16_H_18_O_9_	354.09508	0.24	353.08783	192.05876; 191.05544; 173.04474; 127.03876; 85.02806
Caffeic Acid	8.71	C_9_H_8_O_4_	180.04226	0.37	179.03501	135.04390; 107.04881
Ferulic Acid	14.98	C_10_H_10_O_4_	194.05791	0.62	193.05066	178.02635; 149.05974; 134.03615; 106.0424
Ellagic Acid	14.44	C_14_H_6_O_8_	302.00627	1.62	300.99899	185.02349; 283.98961; 229.01391; 157.01006
Abscinic Acid	15.73	C_15_H_20_O_4_	264.13616	1.42	263.12891	263.12854; 219.13864; 204.11502; 153.09126; 136.05162
*p*-coumaric Acid	10.77	C_9_H_8_O_3_	164.04734	0.18	163.03954	211.0764; 135.00754; 119.0502; 17.0332; 116.0267
Syringic Acid	15.38	C_9_H_10_O_5_	198.05282	0.41	197.04555	182.02049; 123.00697; 166.99693

**Table 2:** The 29 compounds identified from alfalfa and red clover sprouts by UHPLC-Q-Exactive for which the structures were presumed based on high-accuracy analysis of deprotonated precursors and fragment ions of specific components.

Compound Name	R.T. (min)	Formula	Exact Mass	Error (ppm)	Adduct Ion (*m*/*z*)	MS ^2^ Fragments (*m*/*z*)
coumestrol	18.22	C_15_H_8_O_5_	268.03717	1.2	267.0299	266.0373; 239.0487; 211.04058; 167.10689
coumestrol 3-*O*-glucoside	12.46	C_21_H_18_O_10_	430.0900	1.5	429.08274	417.23584; 387.22531; 367.11652; 345.13385; 267.03012
biochanin A	20.09	C_1_6H_12_O_5_	284.06847	1.27	283.06122	269.04132; 268.03809; 267.03015; 239.03487; 224.04756; 212.04695; 211.03928; 195.13850; 154.06250; 132.02031
Sissotrin (biochanin A7-*O*-β-dglucoside)	22.02	C_22_H_22_O_10_	446.1213	1.49	445.11404	269.04587; 283.06130; 268.03778; 166.92354; 131.94290
Prunetine (*O*-methyl genistein)	18.11	C_22_H_12_O_5_	284.06847	1.39	283.06122	269.04132; 268.03809; 267.03015; 240.04300; 239.03487;211.03928
5,7-dihydroxy-2′-methoxyflavone	16.58	C_22_H_12_O_5_	284.06847	1.39	283.06122	269.04584; 268.03799; 267.03015; 240.04300; 239.03473; 223.03952; 211.03963; 148.01559
calycosin (3′-hydroxy-formononetin)	17.52	C_16_H_12_O_5_	284.06847	1.68	283.06122	269.06122; 268.03784; 225.0554; 226.03493; 151.00259; 157.08203; 117.03323; 107.01257
irilone	17.21	C_16_H_10_O_6_	298.04774	0.96	297.04049	269.04590; 252.04297; 178.9951; 133.02837
baptigenin	11.74	C_15_H_10_O_6_	286.04774	1.44	285.04046	269.04565; 240.04242; 136.01556; 109.0282
pseudobaptigenin	21.29	C_16_H_10_O_5_	282.05282	1.36	281.04557	253.05089; 254.05385; 255.14954; 223.02847; 224.04770
pratensein	19.70	C_16_H_12_O_6_	300.06339	0.96	299.05614	284.03284; 283.02505; 257.04111; 135.00754; 211.03932
afrormosin	18.38	C_17_H_14_O_5_	298.08412	1.34	297.07687	282.05362; 283.06802; 267.03021; 253.04797; 167.04965
tectorigenin	18.20	C_16_H_12_O_6_	300.06339	1.41	299.05611	284.03293; 255.03006; 227.03448
alfalone	14.80	C_17_H_14_O_5_	298.08412	1.06	297.07687	281.0450; 269.04120; 211.03958; 135.00395
irisolidone	16.52	C_17_H_14_O_6_	314.07904	1.44	313.07179	298.04849; 269.04581; 255.02989; 211.03957; 165.01836
medicarpin	16.59	C_16_H_14_O_4_	270.08921	1.48	269.08196	254.054408; 253.14426; 141.10812; 117.03334
liquiritigenin	15.30	C_15_H_12_O_4_	256.07356	1.23	255.06631	211.0764; 135.00761; 119.04889; 117.03323
isoliquiritigenin	18.19	C_15_H_12_O_4_	256.07356	1.04	255.06631	211.0764; 135.00760; 119.04889; 117.03323
kaempferol-3-*O*-rutinoside	18.24	C_27_H_30_O_15_	594.15847	1.3	593.15122	299.05615; 284.03281; 255.02997; 227.0341; 229.05032; 133.02834
kaempferol-*O*-glucoside	13.62	C_21_H_20_O_11_	448.10056	1.42	447.09331	284.04077; 284.03299; 255.02995; 243.02979
Isorhamnetin3-*O*-glucoside	15.37	C_22_H_22_O_12_	478.1111	1.43	477.10381	315.04871; 314.04370; 271.02518; 243.03003; 285.04083; 300.02777; 151.00262
ethyl gallate	15.38	C_9_H_10_O_5_	198.05282	0.42	197.04557	181.04961; 169.01326; 151.0031; 121.02814; 107.01214; 83.01234
Luteolin7-glucoside	15.19	C_21_H_20_O_11_	448.10056	1.59	447.09331	287.0359; 286.04431; 285.04077; 227.0359; 199.03964; 151.00264
vitexin (apigenin8-*C*-glucoside)	14.79	C_21_H_20_O_10_	432.10565	1.62	431.09839	341.05179; 339.14789; 269.04587; 240.04268; 225.05542; 197.06077
apigetrin (apigenin-7-glucoside)	14.29	C_21_H_20_O_10_	432.10565	1.78	431.09839	269.0478; 267.03009; 257.08200; 151.00267
chryosoeriol	15.24	C_16_H_12_O6	300.06339	1.29	299.05614	284.03284; 269.04572; 256.0357; 255.05562; 207.08704; 151.00266
chryosoeriol7-glucoside	17.32	C_22_H_22_O_11_	462.11621	1.78	461.10893	446.23111; 289.04852; 283.06140; 255.02997
tricin	18.17	C_17_H_14_O_7_	330.07395	0.68	329.06668	299.05634; 284.03290; 243.03044; 227.03470; 161.02370
azelaic acid	15.11	C_9_H_16_O_4_	188.10486	1.05	187.09761	169.08600; 143.10655; 125.09581; 123.08015; 97.06589

**Table 3:** Key ions in HRMS-MS spectra (*m*/*z* with relative abundances (%) in parenthesis) for the identification of biochanin A and isomers.

Ions	Biochanin A (20.09 min)	Calycosin (17.52 min)	Prunetine (18.11 min)	Glycitein (16.33min)	5,7-dihydroxy-2′-methoxy- flavone (16.58 min)
[M–H]^−^	283.06	283.06	283.06	283.06	283.06
[M-H-CH_3_]^−^	268.03 (97)	268.03 (100)	268.03 (100)	268.03 (100)	268.03 (100)
[M-H-OH]^−^	267.06 (10)	267.06 (4)	267.06 (11)	267.06 (11)	267.06 (18)
[M-H-CO_2_]^−^	-	239.07 (13)	239.07 (6)	239.07 (20)	239.07 (35)
[M-H-CO]^−^	255.06 (25)	255.06 (9)	255.06 (42)	255.06 (12)	255.06 (56)
[M-H-CH_3_-CO]^−^	240.04 (5)	240.04 (10)	240.04(44)	240.04 (29)	240.04 (14)
[M-H-CH_3_-CO_2_]^−^	224.04 (22)	224.04 (100)	224.04 (10)	-	-
[M-H-CH_3_-C_2_H_2_O]^−^	226.04 (12)	226.04 (10)	-	-	-
[M-H-CH_3_-H-CO]^−^	239.03 (10)	239.03 (10)	239.03 (17)	-	239.03 (10)
[M-H-CH_3_-H-CO_2_]^−^	223.04 (16)	223.04 (12)	-	-	-
[M-H-CH_3_-2CO]^−^	212.02 (59)	-	-	212.02 (18)	-
[M-H-CH_3_-CO-CO_2_]	-	-	196.05 (15)	196.05 (62)	
[M-H-CH_3_-CO-H-CO_2_]^−^	195.13 (17)	-	-	-	
[M-H-CO-C-ring]^−^	-	193.05 (18)			
[M-H-CH_3_-CO-H-CO]^−^	211.03 (10)	-	211.03 (6)		-
[M−H−CH_3_−CO−CO_2_−CO]^−^	-	-	-	168 (12)	-
[M-H-CO-B-ring]^−^	-	-	167.03 (23)	167.03 (23)	-
[M-H-CH_3_-CO-B-ring]^−^	147.04 (41)	147.04 (27)	-	147.04 (18)	147.04 (48)
[A-ring fragment]^−^	135.08 (74)	-	-	-	-
[B-ring fragment]^−^	132.02 (48)	132.02 (12)	-	-	-

**Table 4:** Key ions in HRMS-MS spectra (*m*/*z* with relative abundances (%) in parenthesis) for identifying tectorigenin A and isomers.

Ions	Tectorigenin (RT 18.20 min)	Chryosoeriol (RT 15.24 min)	Pratensein (RT 19.70 min)
[M−H]^−^	299.05	299.05	299.05
[M-H-CH_3_]^−^	284.03 (57)	284.03 (21)	284.03 (100)
[M-H-CO]^−^	-	-	271.06 (21)
[M-H-CO-H]^−^	270.05 (100)	-	-
[M-H-CH_3_-OH]^−^	267.02(13)	-	267.02 (48)
[M-CH_3_O]^−^	269.04 (65)	269.04 (100)	-
[M-H-CH_3_-CO]^−^	256.03 (5)	-	256.03 (100)
[M−H-CO_2_]^−^	255.06(24)	255.06 (15)	255.06 (24)
[M-H-C_9_H_6_O_2_]^−^	153.01 (25)	-	-
[M-H-CH3-CO-B-ring]^−^	151.00 (31)	-	-
[M-C_9_H_9_O_3_]^−^	135.00 (34)	135.00 (10)	-

**Table 5:** The results of the quantitative analysis for alfalfa sprouts in µg per g of dried weight vegetal material (alfalfa sprouts samples coded as: ALF – alfalfa, day 1-first day of germination, day 2-second day of germination, day 3-third day of germination, day 4-fourth day of germination, and day 5-fith day of germination).

	µg/g DW	ALF Seeds	ALF Day 1	ALF Day 2	ALF Day 3	ALF Day 4	ALF Day 5
1	catechin	2.16 ± 0.16	2.5 ± 0.18	5.89 ± 0.14	7.53 ± 0.51	NF ^*^	NF
2	caffeic acid	NF	NF	NF	NF	NF	NF
3	myricetin	117 ± 4.6	109.5 ± 6.05	113.8 ± 3.6	214 ± 5.6	62.2 ± 1.3	56.3 ± 2.45
4	*p*-cumaric acid	39.24 ± 2.5	30.18 ± 1.2	24.2 ± 0.8	23.2 ± 1.4	22.72 ± 0.9	15.2 ± 0.75
5	syringic acid	25.63 ± 1.04	4.48 ± 0.17	3.87 ± 0.22	3.88 ± 0.09	3.52 ± 0.16	2.8 ± 0.14
6	genistin	2.12 ± 0.06	6.15 ± 0.32	9.28 ± 0.45	4.8 ± 0.05	3.24 ± 0.15	1.04 ± 0.7
7	chlorogenic acid	2.24 ± 0.11	2.3 ± 0.12	2.36 ± 0.09	2.32 ± 0.06	2.32 ± 0.14	2.48 ± 0.07
8	ferulic acid	82.5 ± 4.2	63.9 ± 3.1	39.8 ± 2.04	43.03 ± 2.14	38.29 ± 1.54	50.10 ± 4.01
9	hyperoside	1209.2 ± 10.7	NF	NF	NF	NF	NF
10	isohamnetin	18.58 ± 0.85	26.12 ± 1.2	35.32 ± 1.62	36.12 ± 4.2	40.24 ± 2.87	15.24 ± 1.45
11	rutin	4.36 ± 0.28	3.85 ± 0.15	4.36 ± 0.32	6.96 ± 0.7	5.88 ± 0.14	3.92 ± 0.25
12	gallic acid	NF	NF	NF	NF	NF	NF
13	ellagic acid	7.8 ± 0.55	8.2 ± 0.17	7.15 ± 0.48	7.8 ± 0.84	7.17 ± 0.12	6.90 ± 0.21
14	formononetin	NF	NF	2.04 ± 0.07	133.5±6.2	12.04±0.17	2.24±0.06
15	pinocembrin	0.52 ± 0.02	1.08 ± 0.04	2.48 ± 0.04	2.6 ± 0.02	2.68 ± 0.07	5.12 ± 0.11
16	apigenin	8.35 ± 0.67	10.29 ± 1.62	13.12 ± 0.31	26.57 ± 2.83	19.33 ± 1.63	33.43 ± 1.44
17	pinstrobin	1.52 ± 0.32	1.6 ± 0.4	1.68 ± 0.7	1.76 ± 0.02	2.24 ± 0.014	6.92 ± 0.41
18	kaempferol	328 ± 9.2	162.14 ± 6.8	15.32 ± 1.87	9.2 ± 0.11	8.48 ± 0.15	6.72 ± 0.1
19	hesperetin	14.79 ± 1.02	26.74 ± 1.26	39.2 ± 1.42	366.91 ± 12.3	209.2 ± 11.8	75.9 ± 2.1
20	genistein	NF	20.15 ± 0.95	41.36 ± 1.12	105.8 ± 3.2	27.4 ± 1.05	10.96 ± 0.42
21	naringenin	0.32 ± 0.01	0.41 ± 0.02	0.52 ± 0.02	0.2 ± 0.01	0.4 ± 0.014	0.52 ± 0.011
22	quercitin	1108.64 ± 9.5	836.1 ± 9.9	725 ± 11.2	393.7 ± 7.9	299.6 ± 7.3	138.96 ± 5.3
23	glycitein	NF	10.01 ± 0.32	21.85 ± 1.4	43.69 ± 5.1	5.2 ± 0.23	6.1 ± 0.12
24	daidzin	NF	NF	10.9±0.32	5.2±0.12	NF	NF
25	daidzein	NF	NF	34.44 ± 2.6	53.96 ± 3.45	72.92 ± 2.9	12.44 ± 0.47
26	crysin	1.53 ± 0.4	1.5 ± 0.08	1.34 ± 0.4	1.67 ± 0.07	1.8 ± 0.09	2.0 ± 0.08
27	abiscisic acid	0.61 ± 0.3	0.48 ± 0.2	0.38 ± 0.15	0.32 ± 0.17	0.47 ± 0.15	NF
	Σ polyphenols	2974.60 ± 1.32	1327.97 ± 3.35	1156.48 ± 3.22	1496.27 ± 3.52	846.92 ± 4.94	455.40 ± 5.01

* NF – not found

**Table 6:** The results of the quantitative analysis for red clover sprouts in µg per g of dried weight (DW) vegetal material (red clover sprouts samples coded as: RCV - red clover, day 1-first day of germination, day 2-second day of germination, day 3-third day of germination, day 4-fourth day of germination, day 5-fith day of germination).

	µg/g DW	RCV Seeds	RCV Day 1	RCV Day 2	RCV Day 3	RCV Day 4	RCV Day 5
1	catechin	134.6 ± 5.8	23.17 ± 3.7	17.57 ± 2.6	10.39 ± 1.55	7.57 ± 0.70	7 ± 1.06
2	caffeic acid	16 ± 1.76	NF *	NF	11.88 ± 1.2	NF	NF
3	myricetin	199.5 ± 6.5	67.41 ± 3.9	30.41 ± 1.5	26.98 ± 4.03	44.22 ± 3.2	16.81 ± 1.06
4	*p*-cumaric acid	19.48 ± 1.086	16.48 ± 1.44	14.68 ± 0.95	12.6 ± 0.6	11.44 ± 0.55	11.6 ± 0.28
5	syringic acid	16.11 ± 1.14	8.68 ± 1.5	6.37 ± 0.24	5.48 ± 1.14	3.17 ± 0.17	2.19 ± 0.04
6	genistin	NF	0.76 ± 0.04	12.2 ± 1.04	20.38 ± 1.28	19.31 ± 2.09	11.9 ± 1.10
7	chlorogenic acid	NF	NF	NF	NF	NF	NF
8	ferulic acid	175.48 ± 6.8	67.56 ± 4.6	99.64 ± 7.5	82 ± 5.2	97.12 ± 8.3	101.48 ± 6.9
9	hyperoside	750.9 ± 10.02	196.2 ± 4.6	12.6 ± 0.6	NF	NF	NF
10	isohamnetin	28.6 ± 0.52	56.04 ± 0.12	43.6 ± 0.34	30.52 ± 0.47	88.48 ± 1.28	38.16 ± 0.9
11	rutin	16 ± 0.9	67.78 ± 4.6	71.14 ± 5.02	71.56 ± 3.95	122.68 ± 7.21	140.2 ± 6.95
12	gallic acid	2 ± 0.04	1.2 ± 0.02	1.04 ± 0.01	NF	1.56 ± 0.02	1.64 ± 0.03
13	ellagic acid	9.08 ± 0.06	10.20 ± 1.14	8.21 ± 1.09	7.8 ± 1.11	7.16 ± 1.15	NF
14	formononetin	14.8 ± 1.03	35.68 ± 3.6	134.2 ± 6.2	172.76 ± 8.05	180.12 ± 6.12	141.36 ± 4.8
15	pinocembrin	NF	0.2 ± 0.03	0.2 ± 0.02	0.24 ± 0.02	0.48 ± 0.03	0.28 ± 0.01
16	apigenin	NF	NF	NF	NF	NF	NF
17	pinstrobin	1.6 ± 0.14	1.6 ± 0.12	1.92 ± 0.2	2.4 ± 0.04	3.24 ± 0.15	4.52 ± 0.04
18	kaempferol	78.8 ± 3.8	5.76 ± 2.05	5.48 ± 2.12	5.48 ± 1.5	6.48 ± 1.9	5.48 ± 2.4
19	hesperetin	2824.8 ± 8.5	759.86 ± 7.3	424.83 ± 6.8	343.62 ± 10.05	203.44 ± 6.5	173.95 ± 4.6
20	genistein	28.76 ± 2.06	435.44 ± 4.7	593.44 ± 5.2	607.2 ± 3.9	499.92 ± 4.8	NF
21	naringenin	1.32 ± 0.7	0.16 ± 0.01	0.24 ± 0.03	0.32 ± 0.03	0.28 ± 0.04	NF
22	quercitin	6714 ± 9.54	2105 ± 6.25	1840.84 ± 5.07	1169.17 ± 5.10	1406.05 ± 3.9	1633.61 ± 3.04
23	glycitein	2.49 ± 1.05	1.9 ± 1.01	17.43 ± 0.95	44.54 ± 2.45	43.74 ± 1.90	27 ± 1.05
24	daidzin	NF	94.1 ± 4.3	78.3 ± 3.7	12.4 ± 2.4	NF	NF
25	daidzein	NF	NF	185.2 ± 6.8	220.2 ± 4.2	263.1 ± 3.7	114.5 ± 4.6
26	crysin	1.09 ± 0.1	1.09 ± 0.2	NF	1.10 ± 0.07	1.11 ± 0.09	1.10 ± 0.4
27	abiscisic acid	0.98 ± 0.4	0.17 ± 0.01	0.17 ± 0.02	0.52 ± 0.25	1.00 ± 0.51	0.92 ± 0.32
	Σ polyphenols	11036.40 ± 2.18	2056.45 ± 3.07	3599.72 ± 1.45	2859.55 ± 3.68	3011.68 ± 2.98	2433.72 ± 4.21

* NF – not found

## Supplementary Materials

The following are available online. Figure S1. Influence of different procedures on the extraction yield of the main active compounds. Results are presented as mean (*n* = 3) values ± STDev. Superscripts with different letters indicate significant differences (*p* < 0.05). Figure S2. Total ions current TIC and the extracted chromatograms of the main identified compounds in alfalfa extract on the third day of germination (the chromatograms were extracted from TIC using a 5 ppm mass accuracy window, negative ion mode, full scan, base peak in the range 150–1000 *m*/*z*). Figure S3. Influence of different procedures on the extraction yield of the main active compounds. Results are presented as mean (*n* = 3) values ± STDev. Superscripts with different letters indicate significant differences (*p* < 0.05). Figure S4. Extracted ion chromatogram for *m*/*z* 299.05 in alfalfa sprout on the third day of germination (**A**) and MS-MS spectra of the diagnostic ions of chryosoeriol (**B**), tectorigenin (**C** and **D**), and pratensein (**E**). Figure S5: Results of the calitative screening: variation of the compounds in the samples (alfalfa and red clover sprout samples coded as flow: RCV-red clover, ALF-alfalfa, s-seeds, day 1-first day of germination, day 2-s day of germination, day 3-third day of germination, day 4-fourth day of germination, day 5-fith day of germination). Table S1: UHPLC-MS/MS method validation parameters.

## References

[1] Oh M.M., Rajashekar C.B. (2009). Antioxidant content of edible sprouts: Effects of environmental shocks. J. Sci. Food Agric..

[2] Brajdes C., Bahrim G., Dinica R., Vizireanu C. (2013). Phenolics composition and their biochemical stability confirmation by in vitro gastrointestinal conditions simulation, for a new functional fermented beverage based on sprouted buckwheat. Rom. Biotechnol. Lett..

[3] Goszcz K., Duthie G.G., Stewart D., Leslie S.J., Megson I.L. (2017). Bioactive polyphenols and cardiovascular disease: Chemical antagonists, pharmacological agents or xenobiotics that drive an adaptive response?. Br. J. Pharm..

[4] Benincasa P., Falcinelli B., Lutts S., Stagnari F., Galieni A. (2019). Sprouted grains: A comprehensive review. Nutrients.

[5] Plaza L., de Ancos B., Cano P.M. (2003). Nutritional and health-related compounds in sprouts and seeds of soybean (Glycine max), wheat (Triticum aestivum L.) and alfalfa (Medicago sativa) treated by a new drying method. Eur. Food Res. Technol..

[6] Silva L.R., Pereira M.J., Azevedo J., Gonçalves R.F., Valentão P., de Pinho P.G., Andrade P.B. (2013). Glycine max (L.) Merr., Vigna radiata L. and Medicago sativa L. sprouts: A natural source of bioactive compounds. Food Res. Int..

[7] Grela E.R., Kiczorowska B., Samolińska W., Matras J., Kiczorowski P., Rybiński W., Hanczakowska E. (2017). Chemical composition of leguminous seeds: Part I—content of basic nutrients, amino acids, phytochemical compounds, and antioxidant activity. Eur. Food Res. Technol..

[8] Witkowska H.E., Biały Z., Jurzysta M., Waller G.R. (2008). Analysis of saponin mixtures from alfalfa (*Medicago sativa L*.) roots using mass spectrometry with MALDI techniques. Nat. Prod. Com..

[9] Oleszek W., Stochmal A., Waksmundzka-Hajnos M., Sherma J. (2010). Cap 25. High performance Liquid Chromatography of Triterpenes (including saponins). High Performance Liquid Chromatography in Phytochemical Analysis.

[10] Abbruscato P., Tosi S., Crispino L., Biazzi E., Menin B., Picco A.M., Pecetti L., Avato P., Tava A. (2014). Triterpenoid glycosides from *Medicago sativa* as antifungal agents against Pyricularia oryzae. J. Agric. Food Chem..

[11] Lei Z., Watson B.S., Huhman D., Yang D.S., Sumner L.W. (2019). Large-Scale Profiling of Saponins in Different Ecotypes of *Medicago truncatula*. Front. Plant Sci..

[12] Rafińska K., Pomastowski P., Wrona O., Górecki R., Buszewski B. (2017). Medicago sativa as a source of secondary metabolites for agriculture and pharmaceutical industry. Phytochem. Lett..

[13] Kolodziejczyk-Czepas J. (2016). Trifolium species – The latest findings on chemical profile, ethnomedicinal use and pharmacological properties. J. Pharm. Pharm..

[14] Rietjens I.M., Louisse J., Beekmann K. (2017). The potential health effects of dietary phytoestrogens. Br. J. Pharm..

[15] Budryn G., Grzelczyk J., Pérez-Sánchez H. (2018). Binding of red clover isoflavones to actin as a potential mechanism of anti-metastatic activity restricting the migration of cancer cells. Molecules.

[16] Spanguolo P., Rasini E., Luini A., Legnaro M., Luzzani M., Casareto E., Carreri M., Paracchini S., Marino F., Cosentino M. (2014). Isoflavone content and estrogenic activity of different batches of red clover (Trifolium pretense L.) extracts: An in vitro study in MCF-7 cells. Fitoterapia.

[17] Klejdus B., Vitamvásová-Štěrbová D., Kubáň V. (2001). Identification of isoflavone conjugates in red clover (Trifolium pratense) by liquid chromatography–mass spectrometry after two-dimensional solid-phase extraction. Anal. Chim. Acta.

[18] Vlaisavljević S., Kaurinović B., Popović M., Vasiljević S. (2017). Profile of phenolic compounds in Trifolium pratense L. extracts at different growth stages and their biological activities. Int. J. Food Prop..

[19] Hong Y.H., Wang S.C., Hsu C., Lin B.F., Kuo Y.H., Huang C.J. (2011). Phytoestrogenic compounds in alfalfa sprout (Medicago sativa) beyond coumestrol. J. Agric. Food Chem..

[20] Goławska S., Łukasik I., Kapusta I., Janda B. (2012). Do the contents of luteolin, tricin, and chrysoeriol glycosides in alfalfa (Medicago sativa L.) affect the behavior of pea aphid (Acyrthosiphon pisum)?. Pol. J. Environ. Stud..

[21] Wang Z., Qu Y., Wang L., Zhang X., Xiao H. (2016). Ultra-high performance liquid chromatography with linear ion trap-Orbitrap hybrid mass spectrometry combined with a systematic strategy based on fragment ions for the rapid separation and characterization of components in Stellera chamaejasme extracts. J. Sep. Sci..

[22] Sun Z., Zuo L., Sun T., Tang J., Ding D., Zhou L., Zhang X. (2017). Chemical profiling and quantification of XueBiJing injection, a systematic quality control strategy using UHPLC-Q Exactive hybrid quadrupole-orbitrap high-resolution mass spectrometry. Sci. Rep..

[23] Zomer P., Mol H.G. (2015). Simultaneous quantitative determination, identification and qualitative screening of pesticides in fruits and vegetables using LC-Q-Orbitrap™-MS. Food Addit. Contam. Part A.

[24] Elmiger M.P., Poetzsch M., Steuer A.E., Kraemer T. (2018). Parameter Optimization for Feature and Hit Generation in a General Unknown Screening Method—Proof of Concept Study Using a Design of Experiment Approach for a High Resolution Mass Spectrometry Procedure after Data Independent Acquisition. Anal. Chem..

[25] Gholami A., De Geyter N., Pollier J., Goormachtig S., Goossens A. (2014). Natural product biosynthesis in Medicago species. Nat. Prod. Rep..

[26] Zhang Y.M., Yan S.J., Cao Z.Z., Shi S.L. (2008). Methodological Study for Total Flavonoid Extraction from Alfalfa by Microwave Assistance. Acta Agrestia Sin..

[27] Kang J., Hick L.A., Price W.E. (2007). A fragmentation study of isoflavones in negative electrospray ionization by MSn ion trap mass spectrometry and triple quadrupole mass spectrometry. Rapid Commun. Mass Spectrom..

[28] Ben Said R., Hamed A.I., Mahalel U.A., Al-Ayed A.S., Kowalczyk M., Moldoch J., Stochmal A. (2017). Tentative characterization of polyphenolic compounds in the male flowers of Phoenix dactylifera by liquid chromatography coupled with mass spectrometry and DFT. Int. J. Mol. Sci..

[29] Raju K.S.R., Kadian N., Taneja I., Wahajuddin M. (2015). Phytochemical analysis of isoflavonoids using liquid chromatography coupled with tandem mass spectrometry. Phytochem. Rev..

[30] Schmidt J. (2016). Negative ion electrospray high-resolution tandem mass spectrometry of polyphenols. J. Mass Spectrom..

[31] Frański R., Gierczyk B., Kozik T., Popenda Ł., Beszterda M. (2019). Signals of diagnostic ions in the product ion spectra of [M − H]^−^ ions of methoxylated flavonoids. Rapid Commun. Mass Spectrom..

[32] Ablajan K. (2011). A study of characteristic fragmentation of isoflavonoids by using negative ion ESI-MSn. J. Mass Spectrom..

[33] Zhao X., Zhang S., Liu D., Yang M., Wei J. (2020). Analysis of Flavonoids in Dalbergia odorifera by Ultra-Performance Liquid Chromatography with Tandem Mass Spectrometry. Molecules.

[34] Liu J., Luo L., Zhang H., Jia B., Lu J., Li P., Chen J. (2015). Rapid screening for novel antioxidants in Glycyrrhiza inflata using high-resolution peak fractionation. J. Funct. Foods.

[35] Bhat G., Shawl A.S., Shah Z., Tantry M. (2014). HPLC-DAD-ESI-MS/MS identification and characterization of major constituents of Iris crocea, Iris germanica and Iris spuria growing in Kashmir Himalayas, India. J. Anal. Bioanal. Tech..

[36] Zhang W.D., Qi L.W., Yang X.L., Huang W.Z., Li P., Yang Z.L. (2008). Identification of the major metabolites of tectorigenin in rat bile by liquid chromatography combined with time-of-flight and ion trap tandem mass spectrometry. Rapid Commun. Mass Spectrom..

[37] Barreira J.C., Visnevschi-Necrasov T., Nunes E., Cunha S.C., Pereira G., Oliveira M.B.P. (2015). Medicago spp. as potential sources of bioactive isoflavones: Characterization according to phylogenetic and phenologic factors. Phytochemistry.

[38] Stochmal A., Simonet A.M., Macias F.A., Oleszek W. (2001). Alfalfa (Medicago sativa L.) flavonoids. 2. Tricin and chrysoeriol glycosides from aerial parts. J. Agric. Food Chem..

[39] Mattioli S., Dal Bosco A., Martino M., Ruggeri S., Marconi O., Sileoni V., Falcinelli B., Castellini C., Benincasa P. (2016). Alfalfa and flax sprouts supplementation enriches the content of bioactive compounds and lowers the cholesterol in hen egg. J. Funct. Foods.

[40] Gatouillat G., Alabdul Magid A., Bertin E., Okiemy-Akeli M.G., Morjani H., Lavaud C., Madoulet C. (2014). Cytotoxicity and apoptosis induced by alfalfa (Medicago sativa) leaf extracts in sensitive and multidrug-resistant tumor cells. Nutr. Cancer.

[41] Barlas N., Özer S., Karabulut G. (2014). The estrogenic effects of apigenin, phloretin and myricetin based on uterotrophic assay in immature Wistar albino rats. Toxicol. Lett..

[42] Kumar V., Rani A., Dixit A.K., Bhatnagar D., Chauhan G.S. (2009). Relative changes in tocopherols, isoflavones, total phenolic content, and antioxidative activity in soybean seeds at different reproductive stages. J. Agric. Food Chem..

[43] Beck V., Rohr U., Jungbauer A. (2005). Phytoestrogens derived from red clover: An alternative to estrogen replacement therapy?. J. Steroid Biochem. Mol. Biol..

[44] Chen K., Li G.J., Bressan R.A., Song C.P., Zhu J.K., Zhao Y. (2019). Abscisic acid dynamics, signaling and functions in plants. J. Integr. Plant Biol..

[45] Jing C.L., Dong X.-F., Tong J.-M. (2015). Optimization of ultrasonic-assisted extraction of flavonoid compounds and antioxidants from alfalfa using response surface method. Molecules.

[46] ICH Harmonised Tripartite Guideline. Validation of Analytical Procedures: Text and Methodology Q2 (R1). Proceedings of the International Conference on Harmonization.

