# Supplementary material for: Comparison of the Polyphenolic Profile of Medicago sativa L. and Trifolium pratense L. Sprouts in Different Germination Stages Using the UHPLC-Q Exactive Hybrid Quadrupole Orbitrap High-Resolution Mass Spectrometry
Source: Molecules. 2020 May 15;25(10):2321. doi: 10.3390/molecules25102321 (PMC7288055; doi:10.3390/molecules25102321)
Supplement: Supplementary file 1 [file molecules-25-02321-s001.pdf]

molecules

MDPI

Article

Comparison of the Polyphenolic Profile of *Medicago sativa l.* and *Trifolium pratense l.* Sprouts in Different Germination Stages Using the UHPLC-Q Exactive Hybrid Quadrupole Orbitrap High-Resolution Mass Spectrometry

Elena Roxana Chiriac [1,2], Carmen Lidia Chiţescu [2,*], Daniela Borda [3], Mariana Lupoae [2], Cerasela Elena Gird [1], Elisabeta-Irina Geană [4], Giorgiana-Valentina Blaga [3] and Rica Boscencu [1]

[1] Faculty of Pharmacy, "Carol Davila" University of Medicine and Pharmacy of Bucharest, 37 Dionisie Lupu Street, Sector 2, 020021 Bucharest, Romania; roxana.chiriac10@yahoo.com (E.R.C.); cerasela.gird@umfcd.ro (C.E.G.); rica.boscencu@umfcd.ro (R.B.)
[2] Faculty of Medicine and Pharmacy, "Dunarea de Jos" University of Galaţi, 35 A.I. Cuza Str., 800010 Galaţi, Romania; mariana.lupoaie@gmail.com
[3] Faculty of Food Science and Engineering, "Dunarea de Jos" University of Galaţi, Str. Domnească 111, 800201 Galaţi, Romania; daniela.borda@ugal.ro (D.B.); giorgianablaga89@ymail.com (G.-V.B.)
[4] National Research &Development Institute for Cryogenics and Isotopic Technologies (ICSI Rm. Valcea), 4th Uzinei Street, 240050, Râmnicu Vâlcea, Romania; irina.geana@icsi.ro
* Correspondence: carmen.chitescu@ugal.ro

Academic Editor: Ping-Chung Kuo
Received: 19 April 2020; Accepted: 14 May 2020; Published: date

Supplementary Materials

1. Optimisation of polyphenols extraction

Ultrasonic assisted extraction (UAE) technique using common solvents as ethanol:water mixtures at elevated temperatures turned out to be very effective in extraction of polyphenols from *Medicago sativa* [1]. In the study referred to, Jing described the optimized extraction conditions for UAE was obtained using response surface method. Ethanol water mixture (50:50) and a temperature of 62 °C was recommended. Using a mixture of ethanol and water on the recoveries of polyphenols from different plants has been discussed in some studies as well [2]. Moreover, ethanol is environmentally-friendly solvents and it is recognized as safe according to the European Food Safety Authority (EFSA) and FAO/WHO Expert Committee on Food Additives [3].

In the present study, three different extraction procedures are tested and compared. Tinctures represent the conventional solid-liquid extraction method to obtain active compounds from plant matrices. Within the technological advances, a broad spectrum of solid-liquid procedures is available including microwave-assisted (MAE) and ultrasound assisted extraction (UAE). The choice of the main variable conditions to be applied within the selected techniques was done considering the literature [4,5,6].

The ratio of dried weight matter (DW) and the extraction solvent was 1:10 *g/g* DW ethanol 70% for both tincture and UAE. A ratio of 1:25 *g/g* DW ethanol 50% was used for MAE. All extractions were performed with hot solvent (60°C for tincture and UAE and 50 °C for MAE). The extraction time was 60 min for UAE and 10 days for tincture. Compared with other techniques, MAE is a

rapid extraction technique which requires irradiation time of seconds [7,8]. An extraction time of 5 minutes by microwave treatment at 300 W power and 50 °C was used.

In the present study it was experimentally demonstrated the superiority of ultrasonic extraction compared to conventional extraction and MAE for the extraction of phenolic compounds from sprouts of alfalfa and red clover. The results indicated that UAE allowed the higher number of compounds in appreciably higher quantities (Figure 1).

2. Optimization of UPLC and MS conditions

Mobile phase and flow rate were investigated in order to obtain the optimal elution conditions for the separation of the analytes. According to the previous reported, methanol was chosen as the organic part of the mobile phase [9]. A small of addition of an organic acid prevent tailoring on the C18 column of phenolic hydroxyl moiety [9]. Polyphenolic compounds generally showed better mass spectrometric responses in negative ionization [9,10]. Therefore, 0.05% and 0.1% formic acid aqueous solution were tested as the mobile phase A with a flow rate of 0.4 mL/min. based on the results the 0.05% formic acid aqueous was preferred as mobile phase A. The elution gradient was progressively slowed to increase the number of data points per chromatographic peak.

Considering our experience and previous studies [10], the heated electrospray ionization parameters were optimized to obtain high sensitivity for most compounds. Optimal conditions were presented in the material and method section.

Operating in vDIA mode the instrument alternates between MS and MS2 scans, and a sufficient number of data points across a chromatographic peak are necessary to ensure a high number of detected peaks. For the Orbitrap, the higher the resolution is set, the slower the scan rate is, even if the effective scan rate also depends on other settings (e.g., AGC intensity and ion accumulation time). Therefore, for the MS scan the resolution was set at 70,000 (FWHM at *m*/*z* 200), AGC target 3e6 and the injection time 200 ms and for the MS2 scan, at 35,000 AGC target 1e6 and the injection time 100 ms; the mass error was less than 2 ppm.

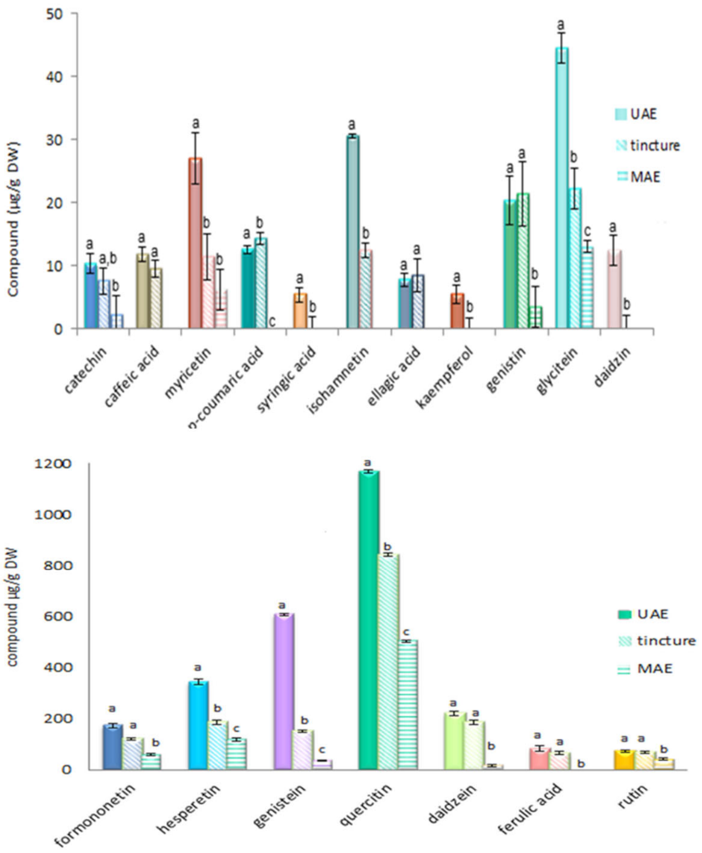

Figure S1. Influence of different procedures on the extraction yield of the main active compounds. Results are presented as mean (n = 3) values ± STDev. Superscripts with different letters indicate significant differences ($p < 0.05$).

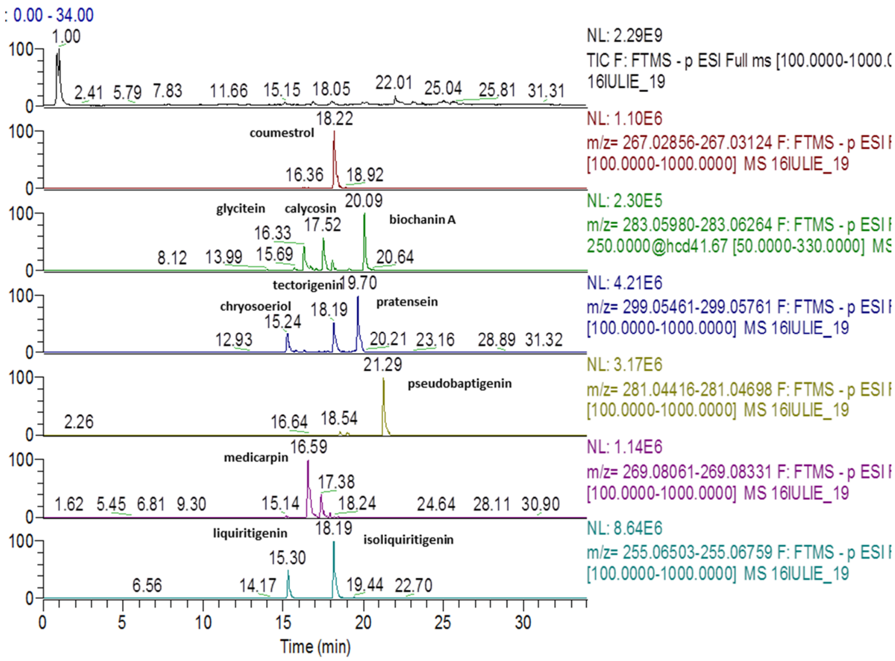

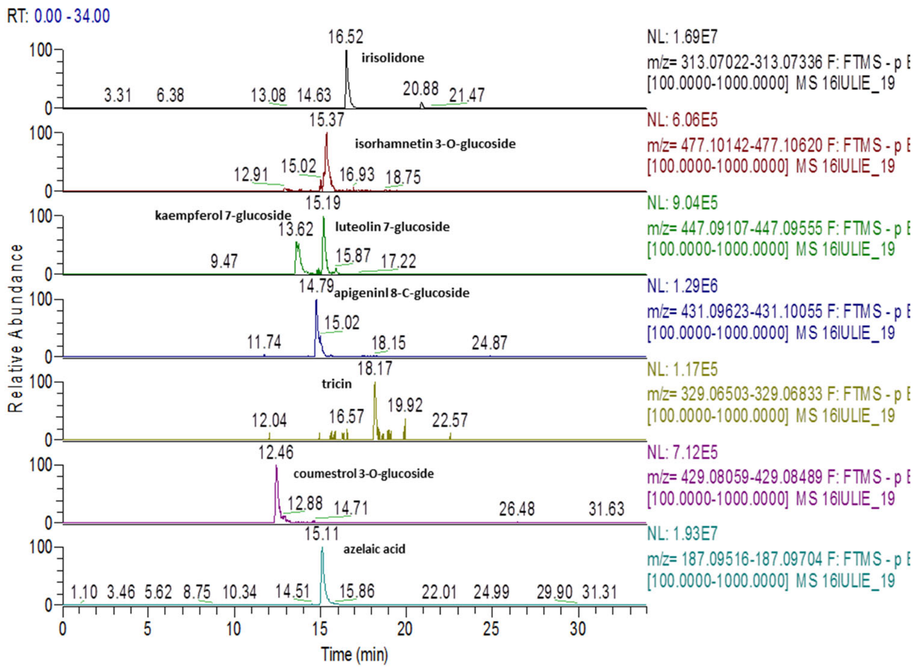

Figure S2. TIC and the extracted chromatograms of the main identified compounds in alfalfa extract in the third day of germination (the chromatograms were extracted from TIC using a 5 ppm mass accuracy window; negative ion mode, full scan, base peak in the range 150–1000 *m*/*z*)

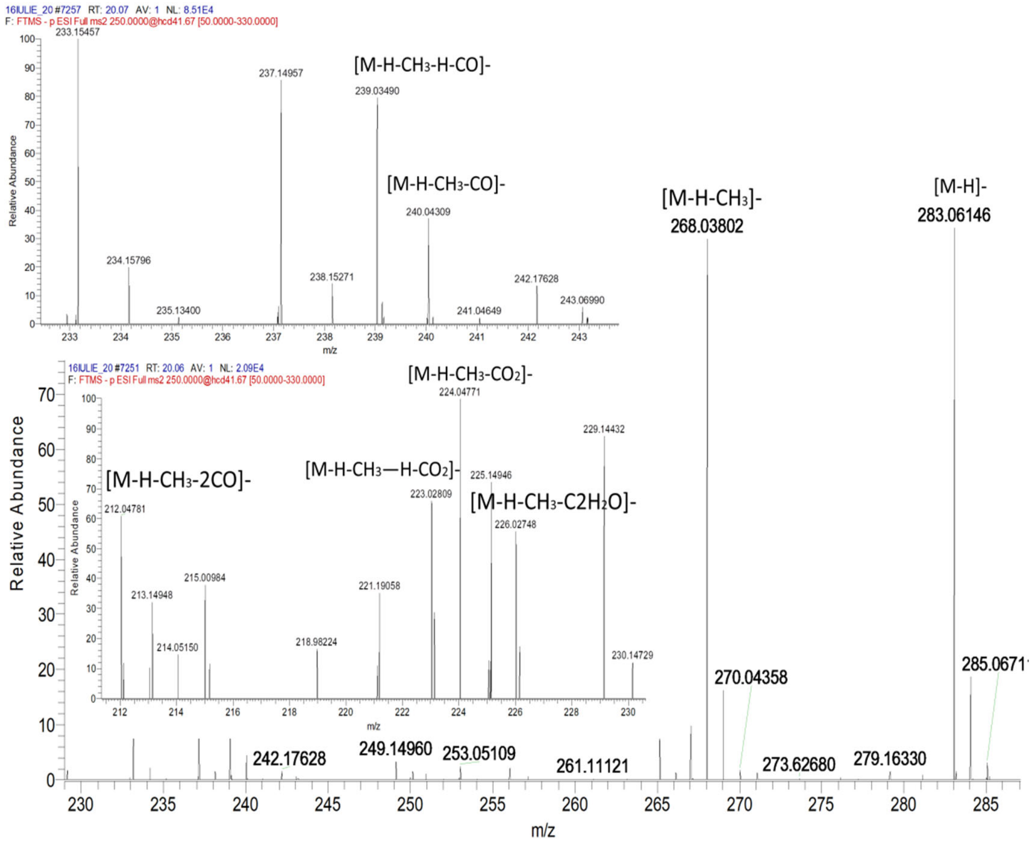

Figure S3. Common product ions spectra of [M-H]- ions of methoxylated isoflavones displayed at *m/z* 283.06 (spectra corresponding to biochanin A in red clover sprouts extract, 3th day of germination).

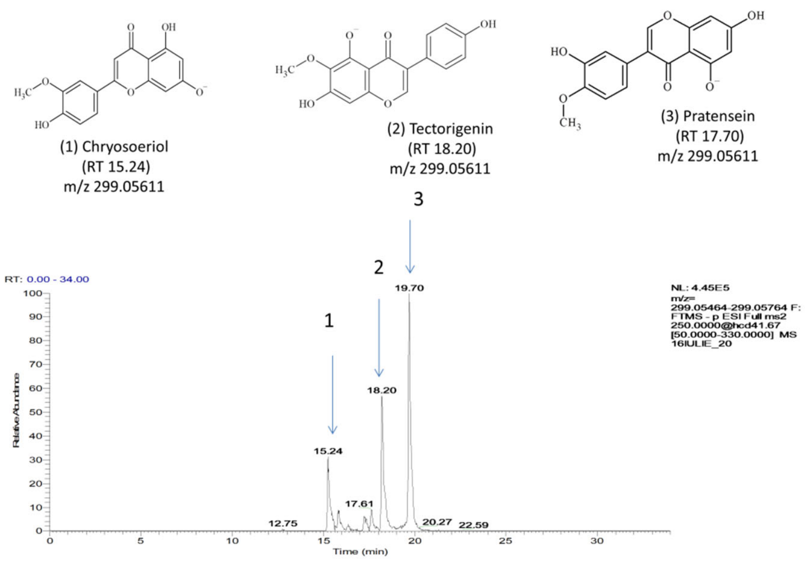

(A)

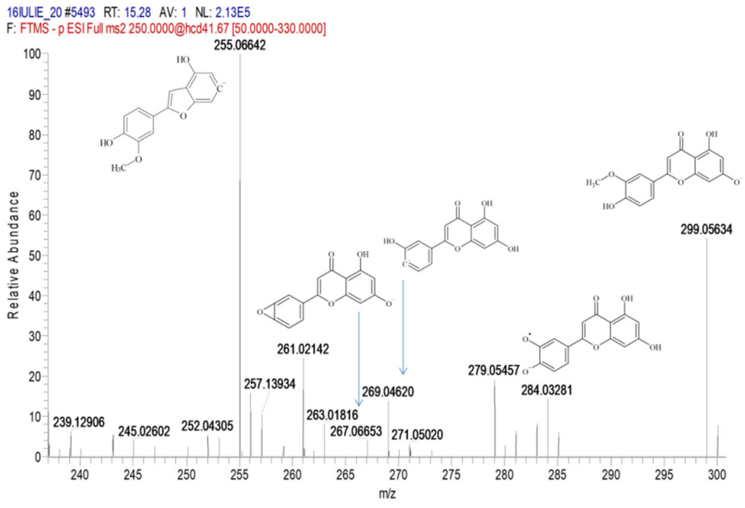

(B)

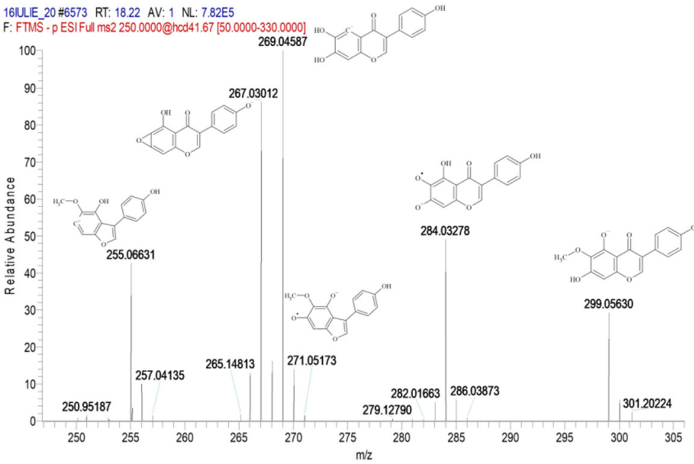
16IULIE_20 #6573 RT: 18.22 AV: 1 NL: 7.82E5
F: FTMS - p ESI Full ms2 250.0000@hcd41.67 [50.0000-330.0000]
Relative Abundance
m/z
269.04587
267.03012
255.06631
284.03278
299.05630
257.04135
265.14813
271.05173
282.01663
286.03873
279.12790
250.95187
301.20224

(C)

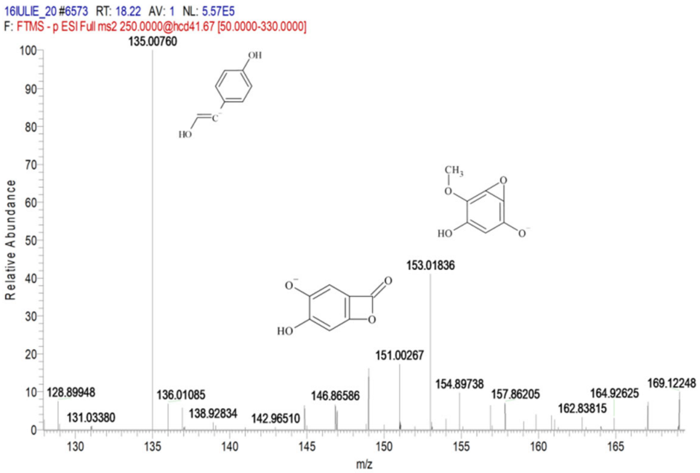
16IULIE_20 #6573 RT: 18.22 AV: 1 NL: 5.57E5
F: FTMS - p ESI Full ms2 250.0000@hcd41.67 [50.0000-330.0000]
Relative Abundance
m/z
135.00760
153.01836
151.00267
128.89948
131.03380
136.01085
138.92834
142.96510
146.86586
154.89738
157.86205
162.83815
164.92625
169.12248

(D)

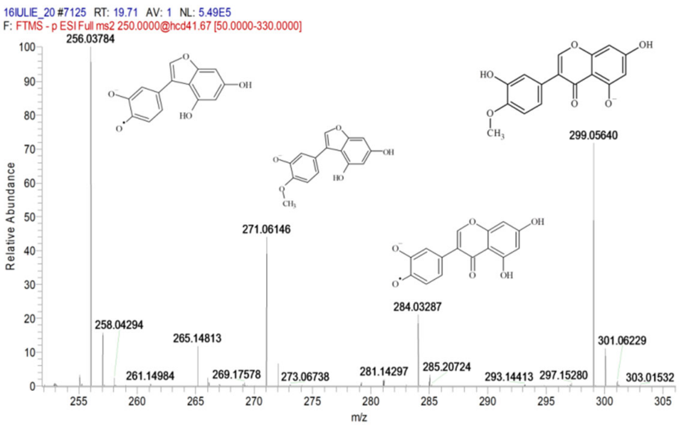
16IULIE_20 #7125 RT: 19.71 AV: 1 NL: 5.49E5
F: FTMS - p ESI Full ms2 250.0000@hcd41.67 [50.0000-330.0000]
Relative Abundance
m/z
256.03784
299.05640
271.06146
284.03287
258.04294
265.14813
261.14984
269.17578
273.06738
281.14297
285.20724
293.14413
297.15280
301.06229
303.01532

(E)

Figure S4. Extracted ion chromatogram for *m*/*z* 299.05 in alfalfa sprout in the 3th day of germination (**A**) and MS-MS spectra of the diagnostic ions of chryosoeriol (**B**), tectorigenin (**C** and **D**) and pratensein (**E**).

	Compounds	RT	ALF seeds	ALF day 1	ALF day 2	ALF day 3	ALF day 4	ALF day 5	RCV seeds	RCV day 1	RCV day 2	RCV day 3	RCV day 4	RCV day 5
1	coumestrol	18.22	x	x	x	x	x	x						
2	coumestrol 3-O-glucoside	12.46	x	x	x	x	x	x						
3	biochanin A	20.09		x	x	x	x	x	X	x	x	x	x	x
4	sissotrin	22.02	x	x	x	x	x				x	x		
5	calycosin	17.52		x	x	x	x	x	x	x	x	x	x	x
6	prunetin (O-methyl genistein)	18.11			x	x	x	x	x	x	x	x	x	x
7	liquiritigenin	15.30	x	x	x	x	x	x	x	x	x		x	x
8	isoliquiritigenin	18.19	x	x	x	x	x	x					x	x
9	irilone	17.21								x	x			
10	pseudobaptigenin	21.29	x	x	x	x	x	x	x	x	x	x	x	x
11	baptigenin	11.74							x	x	x	x	x	x
12	pratensein	19.70		x	x	x	x	x	x	x	x	x	x	x
13	kaempferol-3- O-rutinoside	18.24	x	x	x	x	x	x	x	x	x	x	x	x
14	kaempferol-O-glucoside	13.62	x			x	x	x	x				x	x
15	luteolin 7-O-glucoside	15.19	x	x	x	x	x	x		x	x	x	x	x
16	azelaic acid	15.11	x	x	x	x	x	x	x	x	x	x	x	x
17	ethyl gallate	15.38	x	x	x	x	x	x	x	x	x	x	x	
18	vitexin (apigenin 8-C-glucoside)	14.79	x	x	x	x	x					x	x	
19	apigetrin (apigenin -7-glucoside)	14.29									x	x	x	
20	chrysoeriol	15.24	x	x	x	x	x	x	x		x	x	x	x
21	chrysoeriol 7-glucoside	17.32	x	x	x	x	x	x	x	x	x	x	x	x
22	medicarpin	16.59	x	x	x	x	x	x		x		x	x	x
23	alfalone	14.80									x	x	x	x
24	irisolidone	16.52	x	x	x	x	x	x				x	x	x
25	tricin	18.17	x	x	x	x	x							
26	afrormosin	18.38										x	x	x
27	tectorigenin	18.20		x	x	x	x	x						
28	isorhamnetin 3-O-glucoside	15.37	x	x	x	x	x	x	x	x	x	x	x	x
29	5,7-Dihydroxy-2'-methoxyflavone	16.58					x	x				x	x	x

x	compound detected
	compound not detected

Figure S5: Results of the qualitative screening: variation of the compounds in the samples (alfalfa and red clover sprouts samples coded as flow: RCV — red clover; ALF — alfalfa; s — seeds; day 1-first day of germination; day 2-second day of germination; day 3-third day of germination; day 4-fourth day of germination; day 5-fith day of germination)

Quantitative method validation

Mix standard stock solutions containing 30 analytes were prepared and diluted to six appropriate concentrations for the construction of the calibration curves in the concentration range of 25 to 2500 ng/mL. Each solution was injected in duplicate. Linear regression equation and coefficient of determination (R^2) was obtained. For the applied method, satisfactory linearity and correlation coefficient were achieved with the mentioned linear ranges. The R^2 coefficient for all standards were higher than 0.99, showing a good linearity. RSD values of the intra-day and inter-day of the target compounds were all less than 5.0%, which showed good precision. The values of LODs and LOQs were in the range of 0.15–10.5 μg/g DW and 0.30–35 μg/g DW, respectively (Table S1).

Table S1. UHPLC-MS/MS method validation parameters.

**Compounds/ Validation parameters**	**Calibration equations**	**R^2**	**LOD μg/g DW**	**LOQ μg/g DW**	Precision (RDS, %)		Repetability (RSD, %)
					intra-day	inter-day	
**catechin**	**y = 824313.8x+203017.7**	**0.991**	**0.40**	**1.30**	3.15	2.85	3.21
epicatechin	y = 230287.5x-168641.6	0.992	0.85	2.83	1.41	2.62	2.16
caffeic acid	y = 781534.3x+393060	0.990	1.84	6.13	2.36	4.1	3.82
myricetin	y = 32881x-738887	0.994	1.21	4.03	0.89	1.36	2.07
p-coumaric acid	y = 82377489.4x+486112	0.995	0.62	2.06	1.23	3.04	2.95
syringic acid	y = 72565.8x-82305	0.993	0.35	1.16	2.81	4.01	3.87
genistin	y = 18732x-110805	0.998	1.65	5.50	1.65	1.85	2.04
chlorogenic acid	y = 174329.1x+19222.5	0.998	0.71	2.30	3.45	3.89	4.01
ferullic acid	y = 121050x-2321515	0.999	0.15	0.50	1.25	1.87	2.56
hyperoside	y = 117573.7x+1512847.8	0.998	0.47	1.56	3.21	4.32	3.58
isohamnetin	y = 3022x+156072	0.999	1.02	3.40	2.47	2.67	2.12
rutin	y = 102426.9x+77215.4	0.991	0.65	2.16	2.43	3.16	3.04
gallic acid	y = 449675.5x-332050.7	0.994	0.31	1.03	1.26	2.08	2.74
ellagic acid	y = 10520x-1437986	0.990	1.03	3.04	1.11	1.15	1.27
formononetin	y = 2628478x+16100393	0.999	0.42	1.40	1.48	1.58	2.23
ononin	y =	0.99	10.5	35.1	3.04	4.11	3.98

	211703.5x+1291087	2					
pinocembrin	y = 1301012x-4130476	0.999	0.10	0.30	1.95	2.28	2.65
apigenin	Y = 897709x-73484551	0.998	0.22	0.73	1.65	2.9	3.07
galangin	y = 866716.1x+21534478	0.998	0.15	0.47	1.04	1.87	1.95
pinstrobin	y = 695357x-22084171	0.999	0.15	0.50	2.76	3.16	3.82
kaempferol	y = 324023.5x+35109689	0.991	0.30	1.00	3.07	3.65	3.18
hesperetin	y = 1635.3x-120620	0.990	0.95	3.16	2.89	3.07	3.14
genistein	y = 1598102x+5526494	0.999	0.30	1.00	1.58	2.49	2.61
naringenin	y = 818977.8x+1767512	0.994	0.14	0.46	2.8	3.74	3.06
naringin	Y = 73403.1x+53366.8	0.998	6.90	23.0	1.08	2.16	3.85
quercitin	Y = 125092.1x+747968	0.999	1.60	5.33	1.11	2.65	1.85
glycitein	y = 259340.7x+6212357	0.999	0.55	1.83	2.08	3.18	2.74
daidzin	y = 1017.2x-74781	0.997	1.08	3.60	2.65	3.74	4.08
daidzein	y = 2071x+3009.8	0.999	1.15	3.83	1.43	2.65	2.14
crysin	y = 430529x-185238	0.995	0.21	0.70	1.08	2.14	2.68
abiscisic acid	y = 222271x-396923	0.996	0.15	0.37	1.66	1.89	1.54

References

1. Jing, Ch.L.; Dong, X.-F.; Tong, J.-M.; Optimization of ultrasonic-assisted extraction of flavonoid compounds and antioxidants from alfalfa using response surface method. *Molecules* **2015**, *20*, 15550–15571.
2. Garcia-Castello, E.; Rodriguez-Lopez, A.; Mayor, L.; Ballesteros, R.; Conidi, C.; Cassano, A. Optimization of conventional and ultrasound assisted extraction of flavonoids from grapefruit (*Citrus paradisi* L.) solid wastes. *LWT-Food Sci. Technol.* **2015**, *64*, 1114–1122.
3. The Joint FAO/WHO Expert Committee on Food Additives. Toxicological evaluation of some extraction solvents and certain other substances. In *The FAO Nutrition Meetings Report Series 48A*,Proceedings of the Fourteenth Meeting of the Joint FAO/WHO Expert Committee on Food Additives,Geneva, Switzerland, 24 June–2 July 1970.
4. Rafińska, K.; Pomastowski, P.; Wrona, O.; Górecki, R.; Buszewski, B. Medicago sativa as a source of secondary metabolites for agriculture and pharmaceutical industry. Phytochem. Lett, 2017, *20*, 520–539.

5. Caunii, A.; Pribac, G.; Grozea, I.; Gaitin, D.; Samfira, I. Design of optimal solvent for extraction of bio–active ingredients from six varieties of Medicago sativa. *Chem. Cent. J.* **2012**, *6*, 123.
6. Fumic, B.; Jug, M.; Koncic, M.Z. Multi-response optimization of ultrasound-assisted extraction of bioactive components from Medicago sativa L. *Croatica Chemica Acta*, **2017**, *90*, 481–492.
7. Dahmoune, F.; Nayak, B.; Moussi, K.; Remini, H.; Madani, K. Optimization of microwave-assisted extraction of polyphenols from *Myrtus communis* L. leaves. *Food Chem*. **2015**, *166*, 585–595.
8. Raju, K.S.R.; Kadian, N.; Taneja, I.; Wahajuddin, M. Phytochemical analysis of isoflavonoids using liquid chromatography coupled with tandem mass spectrometry. *Phytochem. Rev.* **2015**, *14*, 469–498.
9. La Barbera, G.; Capriotti, A.L.; Cavaliere, C.; Piovesana, S.; Samperi, R.; Chiozzi, R. Z., Laganà, A. Comprehensive polyphenol profiling of a strawberry extract (Fragaria× ananassa) by ultra-high-performance liquid chromatography coupled with high-resolution mass spectrometry. *Anal. Bioanal. Chem.* **2017**, *409*, 2127–2142.
10. Ciucure, C.T.; Geană, E.I. Phenolic compounds profile and biochemical properties of honeys in relationship to the honey floral sources. *Phytochem Anal* **2019**, *30*, 481–492.
